# Metabolic Insights into Neuropsychiatric Illnesses and Ketogenic Therapies: A Transcriptomic View

**DOI:** 10.3390/ijms25158266

**Published:** 2024-07-29

**Authors:** Smita Sahay, Priyanka Pulvender, Madhu Vishnu Sankar Reddy Rami Reddy, Robert E. McCullumsmith, Sinead M. O’Donovan

**Affiliations:** 1 Department of Neurosciences, University of Toledo College of Medicine and Life Sciences, Toledo, OH 43614, USA; 2 Department of Psychiatry, University of Toledo College of Medicine and Life Sciences, Toledo, OH 43614, USA; 3 Neuroscience Institute, ProMedica, Toledo, OH 43614, USA

**Keywords:** neuropsychiatry, severe mental illnesses, schizophrenia, bipolar disorder, major depressive disorder, bioenergetics, ketogenic, transcriptomic, bioinformatics

## Abstract

The disruption of brain energy metabolism, leading to alterations in synaptic signaling, neural circuitry, and neuroplasticity, has been implicated in severe mental illnesses such as schizophrenia, bipolar disorder, and major depressive disorder. The therapeutic potential of ketogenic interventions in these disorders suggests a link between metabolic disturbances and disease pathology; however, the precise mechanisms underlying these metabolic disturbances, and the therapeutic effects of metabolic ketogenic therapy, remain poorly understood. In this study, we conducted an in silico analysis of transcriptomic data to investigate perturbations in metabolic pathways in the brain across severe mental illnesses via gene expression profiling. We also examined dysregulation of the same pathways in rodent or cell culture models of ketosis, comparing these expression profiles to those observed in the disease states. Our analysis revealed significant perturbations across all metabolic pathways, with the greatest perturbations in glycolysis, the tricarboxylic acid (TCA) cycle, and the electron transport chain (ETC) across all three disorders. Additionally, we observed some discordant gene expression patterns between disease states and ketogenic intervention studies, suggesting a potential role for ketone bodies in modulating pathogenic metabolic changes. Our findings highlight the importance of understanding metabolic dysregulation in severe mental illnesses and the potential therapeutic benefits of ketogenic interventions in restoring metabolic homeostasis. This study provides insights into the complex relationship between metabolism and neuropsychiatric disorders and lays the foundation for further experimental investigations aimed at appreciating the implications of the present transcriptomic findings as well as developing targeted therapeutic strategies.

## 1. Introduction

Neuropsychiatric illnesses, specifically schizophrenia, bipolar disorder, and major depressive disorder, represent a significant global health burden, affecting millions of individuals worldwide [1,2]. These disorders are characterized by a complex relationship among genetic, environmental, and neurobiological factors, leading to diverse clinical manifestations and functional impairments such as cognitive and social dysfunction, emotional dysregulation, and mood and sleep disturbances [3,4]. While traditionally viewed as primarily psychiatric conditions, emerging evidence suggests that brain bioenergetic dysfunction may play a fundamental role in disease pathophysiology [5,6,7] *and* that ketogenic interventions may provide therapeutic benefit in individuals suffering from these disorders [8,9,10].

Bioenergetic function refers to the capacity of a biological system to generate substrates that mediate the transfer of energy. Bioenergetic pathways such as glycolysis, the TCA cycle, and oxidative phosphorylation facilitate glucose-based energy production [11]. Various genetic [12,13], postmortem [14,15,16,17,18], and preclinical [19,20] studies provide support for abnormalities of these pathways in neuropsychiatric illnesses. Alternatively, fatty acids provide another form of fuel. They are released from adipose tissue and catabolized via β-oxidation within the mitochondria of hepatocytes, and to a lesser degree, astrocytes, generating acetyl-CoA [21]. During times when glucose cannot be utilized for energy, acetyl-CoA is shunted towards the generation of ketone bodies, which are, in turn, released into circulation from hepatocytes to travel to the brain, internalized via monocarboxylate transporters, and used as substrates for adenosine triphosphate (ATP) [22]. Given the evidence for carbohydrate metabolism disruptions in these disorders, it follows that neurons may not be able to meet their energy demands via glucose-based oxidative phosphorylation. When glucose is scarce, the brain may intermittently switch to metabolizing ketones for ATP [23]. This metabolic switch to a state of ketosis enhances cognition and motor function, reduces oxidative stress in the brain, and prevents neuronal degeneration in animal models of neuropsychiatric disorders [24]. Ketosis may be achieved via a few methods: prolonged fasting, prolonged or intense exercise, or ketogenic interventions via either diet or exogenous ketone supplements.

Emerging evidence from preclinical and clinical studies suggests that ketogenic interventions may exert beneficial effects on brain function and mood regulation, potentially mitigating the common symptoms observed in neuropsychiatric illnesses. One study administered a ketogenic diet in mice and found that this led to an increased lifespan and increased motor and memory functions [25], a finding consistent with those of another study that found that the ketogenic diet reduced mortality and improved memory in aged mice [26]. More specifically, the ketogenic diet effectively restored abnormal schizophrenia-like behaviors across the whole range of symptoms in rodent models of severe mental illness [27,28,29,30]. In animal stress models of depression, key bioenergetic pathways were improved by the ketogenic diet in combination with antidepressants [31]. Another study found that animals who achieved ketosis after a ketogenic diet were more resistant to metabolic stress in a rat model of bipolar disorder compared to rats fed a standard diet [32]. Although clinical trials have not yet demonstrated the efficacy of the ketogenic diet in bipolar disorder or major depressive disorder, a beneficial effect has been suggested by some case reports [8,33]. Several case reports have also noted the utility of the ketogenic diet in reducing the Positive and Negative Syndrome Scale (PANSS) scores in schizophrenia patients [8,34,35,36]. Furthermore, exogenous ketone supplementation, through the administration of ketone esters or ketone salts, has surfaced as a promising therapeutic strategy for inducing ketosis, modulating metabolic pathways, and improving behavioral deficits in various animal models of neuropsychiatric diseases [20,29,37]. 

Despite these advancements, the precise mechanisms underlying metabolic dysfunction in neuropsychiatric illnesses and ketogenic interventions remain incompletely understood. In this study, we aim to address this gap by employing a novel approach at the transcriptomic level. Specifically, we investigate the dysregulation of 12 key metabolic pathways in schizophrenia, bipolar disorder, and major depressive disorder independently at the gene level, utilizing multiple RNA-sequencing (RNA-Seq) and microarray transcriptomic datasets derived from animal model, cell culture, and postmortem studies. We examine the dysregulation of the same gene sets within pathways in rodent or cell culture models of ketosis, comparing the expression profiles to those observed in the disease states. Notably, our comparative analysis of metabolic perturbations across disease and ketogenic datasets has not been previously explored. We propose two main hypotheses: (I) Metabolic genes will exhibit dysregulation across most pathways in severe mental illnesses, and (II) Metabolic genes will show discordant or concordant expression patterns between severe mental illnesses and ketogenic studies, suggesting that ketosis may either reverse the disease signature, at least partially, or enhance favorable metabolic processes in the context of the disease state. Understanding the molecular pathways and gene expression signatures associated with metabolic dysfunction holds promise for advancing our understanding of neuropsychiatric disease etiology and identifying novel therapeutic avenues. 

## 2. Results

### 2.1. Final Pathways

**Figure 1:**
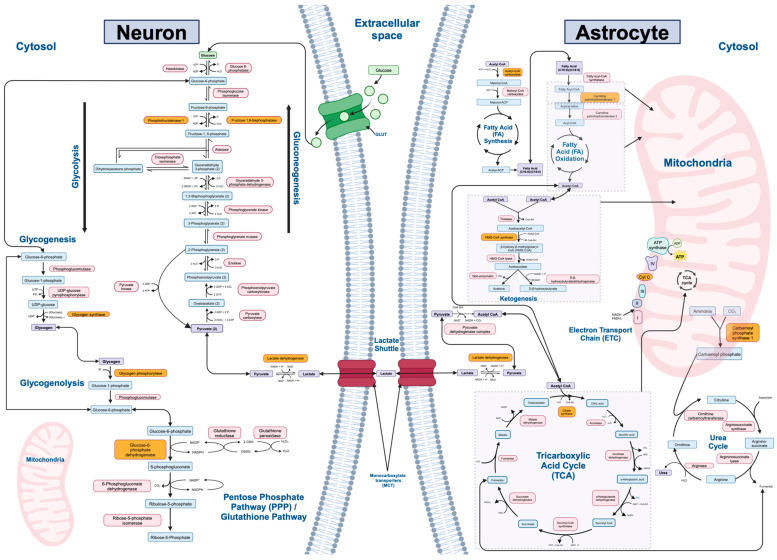
Major metabolic pathways and associated enzymes investigated in the study. This figure illustrates 12 key biochemical pathways involved in energy production, cellular metabolism, and cellular homeostasis: gluconeogenesis, glycolysis, lactate shuttle (between neurons and astrocytes), tricarboxylic acid (TCA) cycle, electron transport chain (ETC), fatty acid synthesis, fatty acid oxidation, glycogenesis, glycogenolysis, urea cycle, and the pentose phosphate/glutathione pathways. Gluconeogenesis, glycolysis, fatty acid synthesis, part of fatty acid oxidation, glycogenesis, glycogenolysis, most of the urea cycle, and the pentose phosphate/glutathione pathways occur in the cytoplasm of cells. Gluconeogenesis may additionally occur in the endoplasmic reticulum. The TCA cycle, ETC, most of fatty acid oxidation, ketogenesis, and part of the urea cycle occurs in the mitochondria within cells. In the brain, the lactate shuttle occurs in the extracellular space between neurons and astrocytes. While ketogenesis and the urea cycle primarily occur in hepatocytes, astrocytes in the brain are also capable of these processes. In the brain, ketogenesis serves as a vital alternative energy source during periods of glucose scarcity or increased metabolic demands. Ketone bodies, such as β-hydroxybutyrate and acetoacetate, are synthesized during prolonged fasting or low carbohydrate intake and may cross the blood–brain barrier to provide fuel for neurons and other brain cells. This metabolic adaptation allows the brain to maintain energy homeostasis and function optimally under varying metabolic conditions. This figure highlights the metabolic pathways and enzymes of interest in the present study, providing a visual representation of the molecular processes under investigation. Enzymes involved in each pathway are shown in pink, metabolites are shown in blue, and the rate-limiting enzyme in each pathway is shown in orange.

Twelve major biochemical pathways involved in mammalian metabolism of macromolecules were identified and utilized in our study: gluconeogenesis, glycolysis, lactate shuttle (between neurons and astrocytes), the tricarboxylic acid (TCA) cycle, the electron transport chain (ETC), fatty acid synthesis, fatty acid oxidation, ketogenesis, glycogenesis, glycogenolysis, the urea cycle, and the pentose phosphate/glutathione pathways (Figure 1). The rate-limiting enzymes, refined gene lists, and number of genes of interest for each metabolic pathway are listed in Table 1.

### 2.2. Transcriptomic Results for Ketosis Datasets

The results after querying and examining the gene sets for each metabolic pathway (Table 1) across the ketosis datasets (*n* = 8) within Kaleidoscope Lookup are reported in Table 2. Column one lists the metabolic pathway. For each pathway, all genes that met the advancement criteria for being significantly upregulated or downregulated in ketosis compared to standard diet treatment groups across datasets are displayed in column four. Of the 242 genes collectively queried across all pathways, 38 genes were significantly dysregulated in ketosis (*p* < 0.05). Column five lists the LFC values of the listed genes by pathway, averaged across all the transcriptomic datasets they were significantly altered in.

The pathways with the highest number of gene perturbations across ketogenic intervention datasets were the ETC (*n* = 14) and the TCA cycle (*n* = 5 genes). The altered genes in the ETC cycle included CYCS, COX4I2, and SDHAF3, which were upregulated (*n* = 3), and ATP5F1C, ATP5F1D, COX411, COX5B, COX6C, CYC1, SDHA, SDHB, NDUFV2, UQCRC1, and UQCRFS1, which were downregulated (*n* = 14). All 14 genes were significantly altered in datasets derived from brain tissue. CYCS, COX5B COX6C, SDHB, and UQCRC1 were additionally significantly altered in liver-derived datasets (Supplementary Table S7). The gene expression level among the upregulated genes (*n* = 3) was approximately 1.3× higher in ketogenic groups compared to groups fed a standard diet across all datasets (LFC = 0.40), whereas the expression level among the downregulated genes (*n* = 11) was approximately 1.5× lower in ketogenic groups compared to groups fed a standard diet (LFC = −0.62).

All genes significantly altered in the TCA cycle were downregulated and included CS, IDH3G, SDHA, SDHB, and SUCLG2 (*n* = 5). All were altered in datasets derived from brain tissue, while SDHB and SUCLG2 were, additionally, altered in liver-derived datasets (Supplementary Table S7). The gene expression level among these five genes collectively was approximately 1.6× lower in ketogenic groups compared to groups fed a standard diet across all datasets (LFC = −0.67).

Notably, aside from the TCA cycle, the pathways that showed no ambiguity in the direction of perturbed genes were glycolysis (*n* = 3, LFC = −0.56) and the pentose phosphate/glutathione pathways (*n* = 3, LFC = −0.73), consisting of only downregulated genes. All significantly altered genes and the associated average LFC values for the remaining metabolic pathways are listed in columns four and five in Table 2.

Although the ETC and TCA cycle exhibited the highest number of gene perturbations, 20% and 17% of these genes, respectively, were significantly dysregulated. The pathways with the highest percentage of overall gene perturbations across ketogenic intervention datasets were glycogenolysis (33%), fatty acid synthesis (30%), and gluconeogenesis (22%). Columns four and five in Table 2 detail the perturbed genes and their associated LFC values in each pathway. Supplementary Table S15 provides the percentage of perturbed genes in each pathway, categorized by the direction of change.

The overall gene expression profile derived for ketogenic intervention datasets across all 12 bioenergetic pathways (columns four and five in Table 2) was compared to the gene expression profiles for schizophrenia, bipolar disorder, and major depressive disorder (columns two and three in Table 2, Table 3 and Table 4, respectively) to assess discordant and concordant expression patterns in disease–ketosis comparisons. 

### 2.3. Transcriptomic Results for Schizophrenia and Schizophrenia–Ketosis Comparison

The results after querying and examining the gene sets for each metabolic pathway (Table 1) across the schizophrenia datasets (*n* = 35) are also reported in Table 2. For each pathway, all genes that met the advancement criteria for being significantly upregulated or downregulated in schizophrenia compared to control treatment groups across datasets are displayed in column two. Of the 242 genes queried, 52% of genes (*n* = 126) were significantly dysregulated in schizophrenia (*p* < 0.05). Column five lists the LFC values of the disrupted genes by pathway, averaged across all the transcriptomic datasets they were significantly altered in.

Across the schizophrenia brain tissue- or cell-derived transcriptomic datasets, the pathways with the greatest number of gene perturbations were the ETC (*n* = 39), TCA cycle (*n* = 14), glycolysis (*n* = 14), lactate shuttle (*n* = 11), and pentose phosphate/glutathione pathways (*n* = 10). Of the significantly altered genes in the ETC (*n* = 39), 35 (90%) were downregulated. The expression level of these genes was collectively approximately 1.2× lower in schizophrenia subject groups compared to non-psychiatrically ill control subject groups (LFC = −0.30). Four (10%) of the significantly altered genes were upregulated, and their expression level was approximately 1.4× higher in schizophrenia compared to control groups (LFC = 0.56). The names of the dysregulated genes within this pathway are listed in Table 2.

In the TCA cycle, seven (50%) of the significantly altered genes (*n* = 14) were upregulated: ACO1, ACO2, CS, DBT, IDH2, SDHA, and SUCLG2. The gene expression level among the upregulated genes was approximately 1.3× higher in schizophrenia compared to control groups (LFC = 0.39). Seven (50%) of the significantly altered genes were also downregulated: DLD, IDH1, IDH3B, MDH2, SDHB, SDHD, and SUCLA2. The expression level among these genes was approximately 1.2× lower in the schizophrenia compared to control groups (LFC = −0.33).

Nine (64%) of the significantly altered glycolytic genes (*n* = 14) were upregulated: ALDOA, ALDOB, ALDOC, GAPDHS, PGAM2, ENO1, ENO3, PKLR, and PKM. The expression level among these genes was approximately 1.5× higher in schizophrenia compared to control groups (LFC = 0.68). Five (36%) of the 14 total significantly altered glycolytic genes were downregulated: GAPDH, PFKM, PFKP, PGK1, and PGK2. The expression level in this group was also approximately 1.5× lower in schizophrenia compared to control groups (LFC = −0.58).

In the lactate shuttle, six (55%) of the significantly altered genes (*n* = 11) were upregulated: GLUL, LDHC, SLC16A3, SLC1A2, SLC1A3, and SLC2A1, which were collectively expressed in schizophrenia groups at a level of 1.5× higher than in control groups (LFC = 0.58). Five (45%) of the 11 total significantly altered genes were downregulated: GLS2, LDHA, SLC1A1, SLC2A11, and SLC2A14. The expression of these genes was approximately 1.2× lower in schizophrenia compared to control groups (LFC = −0.23).

In the pentose phosphate/glutathione pathways, five (50%) of the significantly altered genes (*n* = 10) were upregulated: G6PD, HK2, RPE, and TKT. Five (50%) of the significantly altered genes were downregulated: GCLC, GSS, RPIA, SOD1, and TALDO1. The expression level among the upregulated genes was 1.3× higher (LFC = 0.52), whereas the expression level of the downregulated genes was 1.2× lower (LFC = −0.26), in schizophrenia compared to control groups. All significantly altered genes and associated average LFC values for the remaining metabolic pathways are listed in columns two and three in Table 2.

The pathways with the highest percentage of overall gene perturbations across schizophrenia datasets were ketogenesis (66%), the urea cycle (63%), and fatty acid synthesis (62%), the last of which was also a top perturbed pathway based on the percentage of disrupted genes in ketosis. Supplementary Table S16 provides the percentage of perturbed genes in each pathway, categorized by the direction of change.

Finally, the bioenergetic DGE profile among schizophrenia datasets (*n* = 35) was compared to the bioenergetic DGE profile for ketogenic intervention datasets (*n* = 8) to assess discordant and concordant expression patterns. In the ETC, both discordant and concordant expression patterns were seen between the two groups. The gene SDHA showed a discordant pattern (i.e., it was upregulated in schizophrenia but downregulated in ketosis). The gene SDHAF3 also showed a discordant pattern, as it was downregulated in schizophrenia and upregulated in ketosis. The following genes all showed a concordant expression profile: COX4I2, ATP5F1C, COX5B, COX6C, CYC1, SDHB, NDUFV2, UQCRC1, and UQCRFS1, where COX4I2 was upregulated in both groups and the remaining genes were downregulated in both. In the TCA cycle, the genes CS, SDHA, and SUCLG2 were upregulated in schizophrenia but downregulated in ketosis, and SDHB was downregulated in both. In glycolysis, the gene ENO1 showed a discordant expression pattern between the two groups (i.e., ENO1 was upregulated in schizophrenia and downregulated in ketosis), whereas the genes GAPDH and PFKP showed a concordant expression pattern, being downregulated in both states. In the pentose phosphate/glutathione pathways, G6PD was upregulated in schizophrenia but downregulated in ketosis, whereas RPIA was downregulated in both. In gluconeogenesis, the gene PC was upregulated in both schizophrenia and ketosis. In glycogenolysis, PYGL was upregulated in both. In fatty acid synthesis, DECR1 was upregulated in schizophrenia but downregulated in ketosis, whereas ACACB and HADH were upregulated in both. Lastly, in the urea cycle, OAT was downregulated in both. 

**Table 2:** Gene hits by pathway in schizophrenia vs. ketogenic intervention datasets.

Metabolic Pathway	Significant Genes by Pathway in Schizophrenia (SCZ) Datasets (*n* = 35)	SCZ Average LFC Values	Significant Genes by Pathway in Ketogenic Intervention (KI) Datasets (*n* = 8)	KI Average LFC Values
Gluconeogenesis	Upregulated: *PC*, PCK1	1.08	Upregulated: *PC*	0.23
	Downregulated: G6PC3, PFKFB2	−0.32	Downregulated: PCK2	−1.56
Glycolysis	Upregulated: ALDOA, ALDOB, ALDOC, GAPDHS, PGAM2, **ENO1**, ENO3, PKLR, PKM	0.68		
	Downregulated: *GAPDH*, PFKM, *PFKP*, PGK1, PGK2	−0.58	Downregulated: **ENO1**, *GAPDH*, *PFKP*	−0.56
Lactate Shuttle (Neuron–Astrocyte)	Upregulated: GLUL, LDHC, SLC16A3, SLC1A2, SLCIA3, SLC2A1	0.58	Upregulated: SLC16A1	0.29
	Downregulated: GLS2, LDHA, SLC1A1, SLC2A11, SLC2A14	−0.23		
Tricarboxylic Acid (TCA) Cycle	Upregulated: ACO1, ACO2, **CS**, DBT, IDH2, **SDHA**, **SUCLG2**	0.39		
	Downregulated: DLD, IDH1, IDH3B, MDH2, *SDHB*, SDHD, SUCLA2	−0.33	Downregulated: **CS**, IDH3G, **SDHA**, *SDHB*, **SUCLG2**	−0.67
Electron Transport Chain (ETC)	Upregulated: *COX4I2*, COX6B2, **SDHA**, UQCR10	0.56	Upregulated: CYCS, *COX4I2*, **SDHAF3**	0.40
	Downregulated: ATP5F1A, ATP5F1B, *ATP5F1C*, ATP5F1E, COQ10B, COQ4, COQ7, COX5A, *COX5B*, COX6A1, COX6B1, *COX6C*, COX7A1, COX7A2, COX7B, COX7C, COX8A, *CYC1*, NDUFA12, NDUFA8, NDUFB5, NDUFC2, NDUFS4, NDUFS5, NDUFV1, *NDUFV2*, NDUFV3, **SDHAF3**, *SDHB*, SDHD, UQCR11, UQCRB, *UQCRC1*, *UQCRFS1*, UQCRQ	−0.30	Downregulated: *ATP5F1C*, ATP5F1D, COX411, *COX5B*, *COX6C*, *CYC1*, **SDHA**, *SDHB*, *NDUFV2*, *UQCRC1*, *UQCRFS1*	−0.62
Fatty Acid Synthesis	Upregulated: *ACACB*, **DECR1**, FASN, *HADH*	0.50	Upregulated: *ACACB*, *HADH*	0.70
	Downregulated: ELOVL1, MCAT, OLAH, OXSM	−0.68	Downregulated: **DECR1**, PPT1	−0.69
Fatty Acid Oxidation	Upregulated: ACAA2, CPT1C, CPT2, HADHA, HSD17B10	0.56	Upregulated: HADH	0.41
	Downregulated: SCP2	−0.48		
Ketogenesis	Upregulated: ACAA2, BDH1, BDH2, HMGCLL1	0.82		
	Downregulated: ACAA1, HMGCS1	−0.30		
Glycogenesis	Upregulated: HK2	0.33		
	Downregulated: GBE1, GSK3A, GSK3B, GYS1	−0.22	Downregulated: PGM1	−0.50
Glycogenolysis	Upregulated: *PYGL*	0.54	Upregulated: *PYGL*, PYGM	0.56
	Downregulated: G6PC3	−0.36	Downregulated: PGM1	−0.50
Urea Cycle	Upregulated: ACY1, AGMAT, CAD	0.90		
	Downregulated: ARG2, ASS1, CPS1, *OAT*	−0.32	Downregulated: *OAT*	−0.56
Pentose Phosphate/ Glutathione Pathways	Upregulated: **G6PD**, GPI, HK2, RPE, TKT	0.52		
	Downregulated: GCLC, GSS, *RPIA*, SOD1, TALDO1	−0.26	Downregulated: **G6PD**, GSR, *RPIA*	−0.73

Table includes significant genes that survived correction for multiple comparisons and were dysregulated (either up- or down-regulated) across multiple (at least two) datasets in our Kaleidoscope “Lookup” study (*p* < 0.05). Blank cells indicate no genes were significantly altered. Bolded genes indicate those with discordant gene expression patterns between schizophrenia datasets (*n* = 35) and ketogenic datasets (*n* = 8). Italicized genes indicate those with concordant gene expression patterns between schizophrenia and ketogenic datasets. LFC values indicate the average LFC among the significant gene hits for the indicated pathway in the study. Abbreviations: SCZ—schizophrenia; KI—ketogenic intervention; LFC—Log_2_ fold change.

### 2.4. Transcriptomic Results for Bipolar Disorder and Bipolar Disorder–Ketosis Comparison

Table 3, like Table 2, reports the differentially expressed metabolic genes by pathway and the associated LFC values across the bipolar disorder datasets (*n* = 55). Of the 242 genes queried, 54% of genes (*n* = 130) were significantly dysregulated in bipolar disorder (*p* < 0.05). 

In the brain tissue- or cell-derived bipolar disorder transcriptomic datasets, of the 12 metabolic pathways, the ETC (*n* = 43), glycolysis (*n* = 16), and the TCA cycle (*n* = 15) had the highest number of gene perturbations. In the ETC, 19 (44%) of the significantly altered genes (*n* = 43) were upregulated, with an expression level approximately 1.5× higher in the subjects with bipolar disorder compared to non-psychiatrically ill control subject groups (LFC = 0.64). Of the significantly altered genes, 24 (56%) were downregulated in the ETC. The expression level in this group was also approximately 1.5× lower in bipolar compared to control groups (LFC = −0.57). The names of the dysregulated genes within this pathway are listed in Table 3.

Of the significantly perturbed glycolic genes (*n* = 16), 12 (75%) were downregulated: ALDOA, ENO1, ENO2, GAPDH, GCK, GPI, PFKL, PFKM, PFKP, PGAM4, PGK1, and PKLR. This group’s expression was approximately 1.5× lower in bipolar compared to control groups (LFC = −0.58). Four (25%) of the 16 total significantly altered genes were upregulated: BPGM, HK2, HK3, and PGAM2, with an expression that was 1.7× higher in bipolar compared to control groups (LFC = 0.84).

The TCA cycle showed that 12 (80%) of the significantly altered genes (*n* = 15) were downregulated: ACLY, ACO2, CS, DLAT, IDH2, IDH3B, MDH2, OGDHL, SDHC, and SUCLA2. Their expression level was approximately 1.3× times lower in bipolar compared to control groups (LFC = −0.52). Three (20%) of the 12 total significantly altered genes were upregulated: ACO1, DBT, and IDH1, with their expression also being approximately 1.3× higher in bipolar compared to control groups (LFC = 0.38).

The pathways with the highest percentage of overall gene perturbations across bipolar disorder datasets were the urea cycle (72%), fatty acid synthesis (69%), and the ETC (63%), the former two of which were also among the top pathways by percentage in the schizophrenia cohort. Supplementary Table S17 provides the percentage of perturbed genes in each pathway, categorized by the direction of change.

Again, the bioenergetic DGE profile among bipolar disorder datasets (*n* = 55) was compared to the bioenergetic DGE profile for ketogenic intervention datasets (*n* = 8) to assess discordant and concordant expression patterns. Like the schizophrenia–ketosis ETC analysis, both discordant and concordant expression patterns were observed in the bipolar–ketosis comparison. NDUFV2 was upregulated in bipolar disorder but downregulated in ketosis, whereas CYCS and SDHAF3 were downregulated in bipolar disorder but upregulated in ketosis. COX4I1 was upregulated in both, whereas ATP5F1D, COX5B, CYC1, and UQCRC1 were downregulated in both. In glycolysis, only concordant expression patterns were observed, with ENO1, GAPDH, and PFKP being downregulated in both groups. In the TCA cycle, CS and IDH3G were downregulated in both. In the lactate shuttle, SLC16A1 was upregulated in both states. In fatty acid synthesis, DECR1 was upregulated in bipolar but downregulated in ketosis, whereas PPT1 was downregulated in both. In glycogenolysis, PYGL was upregulated in both. In the urea cycle, OAT was downregulated in both. Lastly, in the pentose phosphate/glutathione pathways, RPIA was upregulated in bipolar disorder but downregulated in ketosis, whereas G6PD was downregulated in both.

**Table 3:** Gene hits by pathway in bipolar disorder vs. ketogenic intervention datasets.

Metabolic Pathway	Significant Genes by Pathway in Bipolar Disorder (BPD) Datasets (*n* = 55)	BPD Average LFC Values	Significant Genes by Pathway in Ketogenic Intervention (KI) Datasets (*n* = 8)	KI Average LFC Values
Gluconeogenesis	Upregulated: FBP2, PCK1	1.08	Upregulated: PC	0.23
	Downregulated: G6PC3	−0.43	Downregulated: PCK2	−1.56
Glycolysis	Upregulated: BPGM, HK2, HK3, PGAM2	0.84		
	Downregulated: ALDOA, *ENO1*, ENO2, *GAPDH*, GCK, GPI, PFKL, PFKM, *PFKP*, PGAM4, PGK1, PKLR	−0.58	Downregulated: *ENO1*, *GAPDH*, *PFKP*	−0.56
Lactate Shuttle (Neuron–Astrocyte)	Upregulated: LDHC, *SLC16A1*, SLC2A1, SLC2A10, SLC2A12	1.06	Upregulated: *SLC16A1*	0.29
	Downregulated: SLC1A2, SLC2A11, SLC2A3	−0.84		
Tricarboxylic Acid (TCA) Cycle	Upregulated: ACO1, DBT, IDH1	0.38		
	Downregulated: ACLY, ACO2, *CS*, DLAT, IDH2, IDH3B, *IDH3G*, MDH2, OGDHL, SDHAF4, SDHC, SUCLA2	−0.51	Downregulated: *CS*, *IDH3G*, SDHA, SDHB, SUCLG2	−0.67
Electron Transport Chain (ETC)	Upregulated: *COX4I2*, COX6A2, COX6B2, COX8C, MT-ATP6, MT-CO1, MT-CO2, MT-CO3, MT-CYB, MT-ND1, MT-ND2, MT-ND3, MT-ND4, MT-ND4L, MT-ND5, MT-ND6, **NDUFV2**, UQCRC2, UQCRH	0.65	Upregulated: **CYCS**, *COX4I2*, **SDHAF3**	0.40
	Downregulated: ATP5F1A, ATP5F1B, *ATP5F1D*, ATP5F1E, COQ10A, COQ4, COQ7, *COX5B*, COX7B2, COX8A, *CYC1*, **CYCS**, NDUFA11, NDUFA12, NDUFB5, NDUFC2, NDUFS1, NDUFV1, **SDHAF3**, SDHAF4, SDHC, UQCR10, UQCR11, *UQCRC1*	−0.57	Downregulated: ATP5F1C, *ATP5F1D*, COX411, *COX5B*, COX6C, *CYC1*, SDHA, SDHB, **NDUFV2**, *UQCRC1*, UQCRFS1	−0.62
Fatty Acid Synthesis	Upregulated: **DECR1**, ELOVL3, OLAH, OXSM	0.90	Upregulated: ACACB, HADH	0.70
	Downregulated: ACACA, SLOVL6, FASN, MCAT, *PPT1*	−0.51	Downregulated: **DECR1**, *PPT1*	−0.69
Fatty Acid Oxidation	Upregulated: CPT2, HADHB, SCP2	0.46	Upregulated: HADH	0.41
	Downregulated: ACSBG2, CPT1C, ECHS1	−0.76		
Ketogenesis	Upregulated: HMGCL, HMGCS2	1.16		
	Downregulated: ACAT1, HMGCLL1, HMGCS1	−1.05		
Glycogenesis	Upregulated: HK2, HK3, UGP2	0.90		
	Downregulated: PGM2	−0.67	Downregulated: PGM1	−0.50
Glycogenolysis	Upregulated: *PYGL*	0.49	Upregulated: *PYGL*, PYGM	0.56
	Downregulated: AGL, G6PC3, PGM2	−0.58	Downregulated: PGM1	−0.50
Urea Cycle	Upregulated: ACY1, ARG1, OTC	0.68		
	Downregulated: ARG2, ASL, ASS1, CAD, *OAT*	−0.45	Downregulated: *OAT*	−0.56
Pentose Phosphate / Glutathione Pathways	Upregulated: GCLC, HK2, HK3, **RPIA**	0.87		
	Downregulated: *G6PD*, GPI, PGD, TKT	−0.61	Downregulated: *G6PD*, GSR, **RPIA**	−0.73

Table includes significant genes that survived correction for multiple comparisons and were dysregulated (either up- or down-regulated) across multiple (at least two) datasets in our Kaleidoscope “Lookup” study (*p* < 0.05). Blank cells indicate no genes were significantly altered. Bolded genes indicate those with discordant gene expression patterns between bipolar disorder (*n* = 55) and ketogenic datasets (*n* = 8). Italicized genes indicate those with concordant gene expression patterns between bipolar disorder and ketogenic datasets. LFC values indicate the average LFC among the significant gene hits for the indicated pathway in the study. Abbreviations: BPD—bipolar disorder; KI—ketogenic intervention; LFC—Log_2_ fold change.

### 2.5. Transcriptomic Results for Major Depressive Disorder and Major Depressive Disorder–Ketosis Comparison

Like Table 2 and Table 3, Table 4 reports the differentially expressed metabolic genes by pathway and the associated LFC values across the major depressive disorder datasets (*n* = 36). Of the 242 genes queried, 58% of genes (*n* = 141) were significantly dysregulated in major depressive disorder (*p* < 0.05).

Across the postmortem major depressive disorder datasets, the greatest pathway disruptions based on the number of perturbed genes were observed in the ETC (*n* = 40), TCA cycle (*n* = 23), glycolysis (*n* = 14), and lactate shuttle (*n* = 12) pathways. Of the total dysregulated genes (*n* = 40), 35 (88%) were upregulated in the ETC, with an expression level approximately 1.5× higher in depression subject groups compared to non-psychiatrically ill control subject groups (LFC = 0.57). Five (12%) of the 40 total ETC perturbed genes were downregulated, with an expression level approximately 1.4× lower in depression compared to control groups (LFC = −0.51). The names of the dysregulated genes within this pathway are listed in column four, Table 4. 

In the TCA cycle, 15 (65%) of the total perturbed genes (*n* = 23) were upregulated, with an expression level approximately 1.3× higher in bipolar compared to control groups (LFC = 0.45). Eight (35%) of the 23 total perturbed genes were downregulated, with an expression level approximately 1.2× lower in bipolar compared to control groups (LFC = −0.33). The names of the dysregulated genes within this pathway are listed in Table 4.

Twelve (86%) of the total dysregulated glycolytic genes (*n* = 14) were upregulated: ALDOA, ENO2, GCK, GPI, HK1, HK3, PFKP, PGAM1, PGAM4, PGK1, PKM, and TPI1. The expression of these genes was approximately 1.5× higher in depression compared to control groups (LFC = 0.59). Two (14%) of the 14 total disrupted glycolytic genes were downregulated: ENO4 and PGK2, and their expression was 2.4× lower in depression groups compared to control groups (LFC = −1.19).

Among the dysregulated lactate shuttle pathway genes (12), eight (67%) were downregulated: GLUL, LDHD, SLC1A2, SLC1A3, SLC2A10, SLC2A11, SLC2A12, and SLC2A3. Their expression level was 1.4× lower in depression groups compared to control groups (LFC = −0.53). Four (33%) of the 12 total disrupted genes were upregulated: GLS, LDHA, SLC16A1, and SLC1A6. Their expression was 1.3× higher in depression compared to control groups (LFC = 0.39).

The pathways with the highest percentage of overall gene perturbations across major depressive disorder datasets were the TCA cycle, ketogenesis, and glycogenolysis. In each pathway, 77% of genes were dysregulated, albeit with different ratios of upregulated to downregulated genes. Ketogenesis and glycogenesis were also among the top perturbed pathways based on percentage in the schizophrenia and ketosis cohorts, respectively. Supplementary Table S18 provides the percentage of perturbed genes in each pathway in the major depressive disorder cohort, categorized by the direction of change.

The bioenergetic DGE profile among major depressive disorder datasets (*n* = 36) was again compared to the bioenergetic DGE profile for ketogenic intervention datasets (*n* = 8) to assess discordant and concordant expression patterns. In the ETC, most dysregulated genes (COX5B, COX6C, CYC1, SDHA, SDHB, UQCRC1, and UQCRFS1) showed a discordant expression pattern, as they were upregulated in major depressive disorder and downregulated in ketosis, whereas CYCS, COX4I1, and SDHAF3 showed a concordant expression pattern, being upregulated in both states. In the TCA cycle, CS, IDH3G, SDHA, and SDHB were upregulated in major depressive disorder and downregulated in ketosis, whereas SUCLG2 was downregulated in both. PFKP showed a discordant expression pattern, as it was upregulated in major depressive disorder but downregulated in ketosis. In the lactate shuttle, SLC16A1 was upregulated in both. In fatty acid synthesis, DECR1 was downregulated in both. In glycogenolysis, PYGM was downregulated in major depressive disorder and upregulated in ketosis, whereas PYGL was upregulated in both. Finally, in the pentose phosphate/glutathione pathways, GSR and RPIA were upregulated in major depressive disorder and downregulated in ketosis.

**Table 4:** Gene hits by pathway in major depressive disorder vs. ketogenic intervention datasets.

Metabolic Pathway	Significant Genes by Pathway in Major Depressive Disorder (MDD) Datasets (*n* = 36)	MDD Average LFC Values	Significant Genes by Pathway in Ketogenic Intervention (KI) Datasets (*n* = 8)	KI Average LFC Values
Gluconeogenesis	Upregulated: G6PC2	0.3	Upregulated: PC	0.23
	Downregulated: G6PC3, PCK1, PFKFB2	−0.68	Downregulated: PCK2	−1.56
Glycolysis	Upregulated: ALDOA, ENO2, GCK, GPI, HK1, HK3, **PFKP**, PGAM1, PGAM4, PGK1, PKM, TPI1	0.59		
	Downregulated: ENO4, PGK2	−1.19	Downregulated: ENO1, GAPDH, **PFKP**	−0.56
Lactate Shuttle (Neuron–Astrocyte)	Upregulated: GLS, LDHA, *SLC16A1*, SLC1A6	0.39	Upregulated: *SLC16A1*	0.29
	Downregulated: GLUL, LDHD, SLC1A2, SLC1A3, SLC2A10, SLC2A11, SLC2A12, SLC2A3	−0.53		
Tricarboxylic Acid (TCA) Cycle	Upregulated: ACO2, **CS**, DLD, FH, IDH3A, IDH3B, **IDH3G**, OGDH, PDHA1, **SDHA**, **SDHB**, SDHC, SDHD, SUCLA2, SUCLG1	0.45		
	Downregulated: ACLY, ACO1, ALDH5A1, DBT, IDH1, IDH2, IREB2, *SUCLG2*	−0.33	Downregulated: **CS**, **IDH3G**, **SDHA**, **SDHB**, *SUCLG2*	−0.67
Electron Transport Chain (ETC)	Upregulated: ATP5F1A, ATP5F1B, ATP5F1E, COQ10A, COQ10B, COQ7, **COX5B**, COX6B1, **COX6C**, COX7A1, COX7A2, COX7B, COX7C, **CYC1**, *CYCS*, MT-CO2, MT-CO3, NDUFA12, NDUFB5, NDUFC2, NDUFS4, NDUFS5, NDUFV1, **SDHA**, *SDHAF3*, **SDHB**, SDHC, SDHD, UQCR11, UQCRB, **UQCRC1**, UQCRC2, **UQCRFS1**, UQCRH, UQCRQ	0.57	Upregulated: *CYCS*, COX4I2, *SDHAF3*	0.40
	Downregulated: COX7B2, MT-ATP6, MT-ATP8, MT-ND2, MT-ND5	−0.51	Downregulated: ATP5F1C, ATP5F1D, COX411, **COX5B**, **COX6C**, **CYC1**, **SDHA**, **SDHB**, NDUFV2, **UQCRC1**, **UQCRFS1**	−0.62
Fatty Acid Synthesis	Upregulated: ELOVL3, MCAT, OLAH	0.24	Upregulated: ACACB, HADH	0.70
	Downregulated: *DECR1*, ELOVL1, FASN	−0.35	Downregulated: *DECR1*, PPT1	−0.69
Fatty Acid Oxidation	Upregulated: ACSBG2, HSD17B10, SLC27A2	0.29	Upregulated: HADH	0.41
	Downregulated: ACAA2, ACSBG1, CPT2, ECI1	−0.39		
Ketogenesis	Upregulated: ACAT1, HMGCLL1	0.62		
	Downregulated: ACAA1, ACAA2, BDH1, BDH2, HMGCS1	−0.35		
Glycogenesis	Upregulated: HK1, HK3, UGP2	0.41		
	Downregulated: ADPGK, GYS1, PGM2	−0.33	Downregulated: PGM1	−0.50
Glycogenolysis	Upregulated: G6PC2, *PYGL*	0.66	Upregulated: *PYGL*, **PYGM**	0.56
	Downregulated: AGL, G6PC3, PGM2, PYGB, **PYGM**	−0.44	Downregulated: PGM1	−0.50
Urea Cycle	Upregulated: ARG2, ASS1, OTC	0.33		
	Downregulated: ACY1, ASL, CPS1	−0.62	Downregulated: OAT	−0.56
Pentose Phosphate/Glutathione Pathways	Upregulated: GCLC, GPI, **GSR**, HK1, HK3, PGD, **RPIA**, SOD1, TALDO1	0.34		
			Downregulated: G6PD, **GSR**, **RPIA**	−0.73

Table includes significant genes that survived correction for multiple comparisons and were dysregulated (either up- or down-regulated) across multiple (at least two) datasets in our Kaleidoscope “Lookup” study (*p* < 0.05). Blank cells indicate no genes were significantly altered. Bolded genes indicate those with discordant gene expression patterns between major depressive disorder datasets (*n* = 36) and ketogenic datasets (*n* = 8). Italicized genes indicate those with concordant gene expression patterns between major depressive disorder and ketogenic datasets. LFC values indicate the average LFC among the significant gene hits for the indicated pathway in the study. Abbreviations: MDD—major depressive disorder; KI—ketogenic intervention; LFC—Log_2_ fold change.

### 2.6. Transcriptomic Results for Medication Analysis

The results, after querying and examining the gene sets for each metabolic pathway (Table 1) across the antipsychotic datasets (*n* = 24) and mood-stabilizer datasets (*n* = 7) within Kaleidoscope Lookup, are reported in Table 5. Column one lists the metabolic pathway. For each pathway, all genes that met the advancement criteria for being significantly upregulated or downregulated in medication- compared to vehicle-treated (control) groups across datasets are displayed in columns two and four. Columns three and five list the LFC values of the listed genes by pathway, averaged across all transcriptomic datasets the genes were significantly altered in for antipsychotics and mood stabilizers, respectively. Of the 242 genes collectively queried across all pathways, 91 genes were significantly dysregulated in the antipsychotics analysis, and 44 genes were significantly dysregulated in the mood stabilizer analysis (*p* < 0.05). 

The pathways with the greatest number of genes whose expression was affected by antipsychotics were the ETC (*n* = 18), the TCA cycle (*n* = 10), and glycolysis (*n* = 10). The names of these genes and the magnitude by which they were affected (i.e., LFC values) are listed in columns two and three in Table 5. The pathways with the greatest percentage of genes whose expression was affected by antipsychotics were fatty acid synthesis, glycogenesis, and the pentose phosphate/glutathione pathways, with 62%, 53%, and 53% of the total dysregulated genes, respectively. Supplementary Table S19 provides the percentage of genes whose expression was altered in each pathway by antipsychotics, categorized by the direction of change.

The pathways with the greatest number of genes whose expression was affected by mood stabilizers were the ETC (*n* = 7), the TCA cycle (*n* = 7), and fatty acid oxidation (*n* = 6), the former two of which were also top pathways with genes significantly affected by antipsychotics. The pathways with the greatest percentage of genes whose expression was affected by mood stabilizers were fatty acid oxidation, gluconeogenesis, and ketogenesis, with 35%, 33%, and 33% of the total dysregulated genes, respectively. Supplementary Table S20 provides the percentage of genes whose expression was altered in each pathway by mood stabilizers, categorized by the direction of change.

**Table 5:** Medication Analysis Summary.

Metabolic Pathway	Significantly Altered Genes in Chronic Antipsychotic-Treated vs. Vehicle-Treated Datasets (*n* = 24)	Average LFC Values	Significantly Altered Genes in Chronic Mood Stabilizer-Treated vs. Vehicle-Treated Datasets (*n* = 7)	Average LFC Values
Gluconeogenesis	Upregulated: PFKFB2, PCK2, G6PC1	0.39		
			Downregulated: PFKFB2, PCK1, PCK2	−0.23
Glycolysis	Upregulated: PKLR, PGK1, PFKM, HK2, ALDOC, ENO2	0.36	Upregulated: ENO3	0.37
	Downregulated: PFKP, GAPDH, ALDOB, GPI	−0.45	Downregulated: PFKP, PKLR	−0.37
Lactate Shuttle (Neuron–Astrocyte)	Upregulated: SLC1A3, SLC1A2, SLC16A3, LDHD	0.37	Upregulated: GLUL, SLC1A3	0.37
	Downregulated: SLC2A3, SLC2A1, GLS, SLC1A6, GLUL	−0.31	Downregulated: GLS2, GLS	−0.36
Tricarboxylic Acid (TCA) Cycle	Upregulated: SUCLG1, SDHB, IDH2, IDH1, ACO1	0.26	Upregulated: SDHB, IDH2, ACO1, SDHAF4	0.35
	Downregulated: SDHAF4, PDHA2, IDH3A, ALDH5A1, ACLY	−0.47	Downregulated: SUCLA2, ALDH5A1, DBT	−0.22
Electron Transport Chain (ETC)	Upregulated: UQCRH, SDHB, NDUFV2, NDUFC1, NDUFB5, COX7C, COX6B2, COX5B, COQ4, COQ10A, SDHAF2	0.24	Upregulated: COQ7, COX7A2, COX6C, SDHB, SDHAF4	0.32
	Downregulated: SDHAF4, COX8A, COX7A1, COX6C, COX5A, COQ10B, MT-ND3	−0.23	Downregulated: NDUFS1, SDAHF2	−0.26
Fatty Acid Synthesis	Upregulated: OXSM, HADH, ELOVL1, DECR1	0.32	Upregulated: FASN	0.33
	Downregulated: PPT2, MCAT, FASN, ELOVL6	−0.44		
Fatty Acid Oxidation	Upregulated: HSD17B10, HADH, ECI1, ECHS1, ACAA2	0.27	Upregulated: HADHB, ECHS1, CPT1C, SLC27A2, ECI1, HADHA	0.28
			
Ketogenesis	Upregulated: HMGCS2, BDH1, ACAT1, ACAA2	0.34		
			Downregulated: HMGCS2, BDH2, HMGCS1	−0.31
Glycogenesis	Upregulated: HK2, GSK3A	0.21	Upregulated: PGM1, PGM2, GSK3B	0.20
	Downregulated: PGM1, PGM2, GSK3B, GBE1, ADPGK	−0.39	Downregulated: GCKR	−0.25
Glycogenolysis	Upregulated: G6PC1	0.20	Upregulated: PGM1, PGM2	0.21
	Downregulated: PGM1, PGM2, AGL	−0.54		
Urea Cycle	Upregulated: ASS1, ASL, AGMAT	0.44	Upregulated: CAD	0.24
	Downregulated: ARG2	−0.18	Downregulated: ASS1	−0.65
Pentose Phosphate/Glutathione Pathways	Upregulated: TKT, TALDO1, SOD1, RPIA, HK2, GSS, GCLC	0.26		
	Downregulated: RPE, GPI	−0.29	Downregulated: GSS, RPIA	−0.37

Gene hits by pathway in chronic antipsychotic datasets (*n* = 24) and chronic mood stabilizer datasets (*n* = 7) derived from animal models. Table includes significant genes that survived correction for multiple comparisons and were significantly altered between medication treatment and control groups across multiple (at least two) datasets in our Kaleidoscope “Lookup” study (*p* < 0.05). Blank cells indicate no genes were significantly altered between medication and control groups. LFC values indicate the average LFC among the significantly altered genes for the indicated pathway in the study. Abbreviations: LFC—Log_2_ fold change.

## 3. Discussion

**Figure 2:**
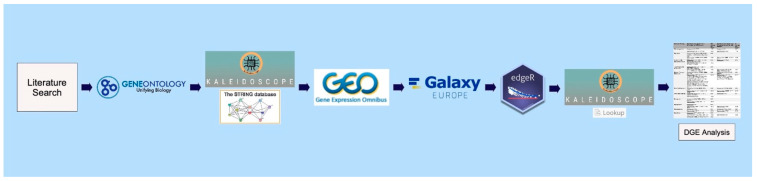
Bioinformatics workflow. This figure illustrates the in silico process utilized to uncover differentially expressed genes across neuropsychiatric illness and ketogenic intervention transcriptomic datasets. First, a literature search was performed to select major biochemical pathways involved in mammalian cellular metabolism. The gene annotation tool, Gene Ontology (GO), was used to obtain the gene symbols associated with enzymes in metabolic pathways. The Search Tool for the Retrieval of Interacting Genes/Proteins (STRING) database within Kaleidoscope was used to refine final gene sets for each pathway. Next, the Gene Expression Omnibus (GEO) data repository was used to filter datasets that assessed the effects of various ketogenic interventions. These datasets were processed utilizing Galaxy and EdgeR and were uploaded to the “ketosis” module within the Kaleidoscope Lookup tool. Finally, gene sets associated with each metabolic pathway were queried within Kaleidoscope Lookup and analysis of the differentially expressed genes was performed among all schizophrenia, bipolar, depression, and ketosis datasets. Side-by-side comparisons of the differentially expressed genes in schizophrenia vs. ketosis, bipolar vs. ketosis, and depression vs. ketosis were performed to assess patterns of dysregulation among pathways for each comparison.

Converging findings in neuropsychiatric illnesses such as schizophrenia, bipolar disorder, and major depressive disorder implicate brain bioenergetic disturbances resulting in disruptions of synaptic signaling, neural circuitry, and neuroplasticity [38]. A growing body of literature also supports the benefit of ketogenic interventions in such disorders. However, the mechanism of metabolic disturbances in these disorders and how ketogenic therapy functions metabolically to aid or circumvent disease pathology remains largely unknown. In the present in silico study, we offer insight into these mechanisms at the transcriptomic level. Utilizing a bioinformatics workflow (Figure 2, Materials & Methods), we confirmed our initial hypotheses. Brain studies employing RNA-Seq and microarrays found significant perturbations in the expression of genes encoding proteins involving 12 core metabolic pathways across severe mental illnesses. Furthermore, many genes exhibited discordant and concordant expression patterns between severe mental illnesses and ketogenic studies, suggesting metabolic modulation at the gene expression level. These findings lay the groundwork for future confirmatory investigations to fully understand the implications of the metabolic mechanisms underlying neuropsychiatric illnesses and ketogenic therapies.

Numerous studies have documented disruptions in bioenergetic pathways across severe mental illnesses such as altered glycolytic enzyme activity [6,39,40,41,42,43,44,45], TCA cycle and ETC enzyme levels [6,7,46,47,48], astrocyte–neuron coupling and lactate metabolism [5,49,50,51], and pentose phosphate pathway and antioxidant metabolism [5,49,52,53]. Our analysis revealed the greatest changes in the expression of genes encoding enzymes in these pathways, complementing existing findings. The greatest significant changes (defined by the significant perturbation of at least 10 genes within a pathway) were observed in glycolysis, the TCA cycle, and the ETC across all three severe mental illnesses. Based on these criteria, significant changes were also observed in the lactate shuttle for schizophrenia and major depressive disorder, as well as in the pentose phosphate/glutathione pathways for schizophrenia. A notable observation was the pronounced perturbation of gene(s) encoding the rate-limiting enzyme in each pathway across all disease profiles. Rate-limiting enzymes are pivotal as they represent inherent bottlenecks, dictating the overall flux through those pathways [54]. Thus, perturbations in the expression of genes encoding rate-limiting enzymes may profoundly influence the activity of the entire pathway, contributing to the metabolic dysfunction observed in these disease states and offering targets for therapeutic intervention.

Several pathways regulate carbohydrate metabolism via sophisticated mechanisms. In times of glucose scarcity, the body may either mobilize stored glucose through glycogenolysis or synthesize glucose from glycogen stores via gluconeogenesis under the hormonal influence of increased glucagon [55,56]. Conversely, increased insulin levels promote glycogenesis, which facilitates the storage of excess glucose as glycogen [57]. In our study, anywhere from *n* = 2 to *n* = 7 genes were perturbed among glycogenolysis, gluconeogenesis, and glycogenesis across disease and ketogenic profiles (Table 2, Table 3 and Table 4). Notably, despite the relatively lower number of genes with altered expression, glycogenolysis was among the top perturbed pathways based on the overall percentage of altered gene expression in the major depressive disorder and ketosis studies, and gluconeogenesis was among the top perturbed pathways in the ketosis study based on the same criteria (Supplementary Tables S15 and S18). Indeed, specific genes exhibited a greater magnitude of expression change than other genes within these pathways; however, these signals, overall, appeared relatively weaker across all cohorts in terms of metabolic perturbations relative to the other pathways investigated. This may be attributed to our study’s focus, which did not fully encompass the hormonal mechanisms that regulate these pathways and may potentially reflect core pathway deficits. For example, disruptions in insulin signaling pathways, which are implicated in neuropsychiatric illnesses, may perturb glycogen synthesis and storage in astrocytes, altering the availability of glucose as a fuel source for neurons [58,59]. Similarly, disrupted glucagon signaling may affect glucose mobilization during periods of stress, further exacerbating energy deficits in the affected brain regions. Further investigations encompassing insulin and glucagon perturbations across these illnesses may capture carbohydrate metabolism deficits and provide additional insights to complement our findings. 

At the core of carbohydrate metabolism lies glycolysis, a major catabolic pathway that occurs in the cytoplasm of cells that utilize and process glucose for energy. Glucose is phosphorylated to glucose-6-phosphate by the enzyme hexokinase (HK) which traps glucose intracellularly to, ultimately, produce circulating carbohydrates in the form of pyruvate. The rate of this pathway is limited by phosphofructokinase (PFK), which catalyzes the irreversible generation of fructose-1,6-bisphosphate. Ultimately, pyruvate, the cell’s primary energy currency ATP, and the reducing agent nicotinamide adenine dinucleotide (NADH) are generated from glucose [60]. One theory suggests that decreased glucose metabolism may trigger chronic inflammatory processes in the brain that aggravate neuronal dysfunction and contribute to neuropsychiatric symptom severity [61]. Another theory states that the mitochondrial dysfunction already prevalent among these disorders may lead to altered glycolytic activity to meet brain energy demands [62,63]. To fully appreciate these relationships, understanding the mechanism driving metabolic changes is a crucial first step. 

Of the 27 glycolytic genes investigated (Table 1), our transcriptomic profiles exhibited perturbation of 51–59% of genes (*n* = 14–16) across severe mental illnesses (Table 2, Table 3 and Table 4). Our primary focus was on discerning whether our data may offer insight into the overall directional alteration of glycolysis within each disorder. In schizophrenia, about twice as many glycolytic genes were upregulated than were downregulated, with a slightly greater magnitude of expression change between schizophrenia and non-psychiatrically ill control groups among upregulated genes compared to downregulated genes (Table 2). However, genes expressing isozymes of the rate-limiting enzyme of glycolysis, PFKM and PFKP, were downregulated, providing support for decreased glycolytic flux in schizophrenia despite the greater number of, and slightly greater magnitude of, expression change among upregulated genes. In bipolar disorder, most glycolytic genes were downregulated, which included PFKM, PFKP, and PFKL, providing support for decreased glycolytic flux, but the magnitude of gene expression change was greater among the upregulated genes (Table 3). In major depressive disorder, a greater number of glycolytic genes were upregulated, which included PFKP, providing support for increased glycolytic flux in major depressive disorder; however, the magnitude of gene expression change was greater among the downregulated genes (Table 4). Overall, these profiles confirm that glycolysis is indeed significantly dysregulated across severe mental illnesses, albeit an interpretation of whether the pathway is predominantly upregulated or downregulated in disease states requires further studies utilizing immunoblotting or protein activity assays of glycolytic targets to understand whether changes at the mRNA level persist at the protein level. Although comprehensive studies assessing all glycolytic enzymes have not been conducted, one study reported a decrease in the gene expression of the glycolytic enzyme PFK1 measured by qPCR in schizophrenia that was consistent with a finding of decreased PFK activity in the same study [45].

Comparing the glycolytic expression profiles between severe mental illnesses and ketogenic datasets revealed a discordant expression pattern in the schizophrenia–ketosis (Table 2) and major depressive disorder–ketosis comparisons (Table 4), where ENO1 was upregulated in schizophrenia, the rate-limiting enzyme PFKP was upregulated in depression, and both were downregulated in ketosis. Considering that ketones are produced from fatty acids in a state of carbohydrate deprivation [60], these findings may indicate that ketones perhaps modulate pathogenic glycolytic expression changes to optimize energy homeostasis via shifting to fatty acid metabolism. The glycolytic profile in ketosis additionally yielded the downregulation of the gene GAPDH (Table 2, Table 3 and Table 4). ENO1, PFKP, and GAPDH were also downregulated in bipolar disorder with a similar magnitude of expression change in both states (Table 3). This concordant expression pattern may, perhaps, be attributed to ketone bodies circumventing a pathogenic energy generation pathway to provide an efficient and sustainable energy supply for the brain via ketone bodies [22]. These findings warrant further studies aimed at investigating the specific metabolic and neurobiological effects of ketogenic therapy and interpreting the mechanistic pathways underlying ketone body effects.

Aside from glycolysis, another fate for glucose-6-phosphate, once sequestered intracellularly, is processing via the pentose phosphate pathway to create ribose-5-phosphate for nucleotide biosynthesis. The enzyme that limits the rate of this pathway is glucose-6-phosphate dehydrogenase (G6PD), a step in which the reducing equivalent nicotinamide adenine dinucleotide phosphate (NADPH) is produced. NADPH aids in fatty acid and cholesterol synthesis and supports the production and regeneration of glutathione, a molecule that is necessary for neutralizing reactive oxygen species and protecting cells from oxidative damage [64,65]. In the central nervous system (CNS), glucose ultimately plays a role in antioxidant replenishment during heightened energetic demands, and the disruption of this pathway is prevalent in the pathophysiology of severe mental illnesses [5,49]. 

In our study, glycolysis was among the top perturbed pathways based on the number of disrupted genes in schizophrenia. The schizophrenia and bipolar disorder analyses showed an equal ratio of upregulated and downregulated genes, and the magnitude of expression change was greater among the upregulated genes; however, the rate-limiting enzyme G6PD was upregulated in schizophrenia (Table 2) and downregulated in bipolar disorder (Table 3). Major depressive disorder data showed significant downregulation of nine genes, albeit with a relatively lower magnitude of expression change between depression and control groups across datasets and no significant expression changes observed with G6PD (Table 4).

The ketosis gene expression profile was partially discordant with schizophrenia (Table 2) and bipolar disorder (Table 3) and entirely discordant with major depressive disorder (Table 4), showing significant downregulation of the genes G6PD, GSR, and RPIA. The upregulation of the pentose phosphate pathway is associated with oxidative stress, disrupted redox signaling, and aberrant cellular proliferation or survival [66,67]. Therefore, the discordant ketogenic expression profile may suggest that these interventions have the capacity to exert a corrective effect on the dysregulated expression of genes in the pentose phosphate and glutathione pathways via attenuating the excessive production of NADPH and ribose-5-phosphate for optimal antioxidant defense mechanisms and fatty acid utilization for energy, although further mechanistic studies are necessary to confirm these transcriptomic-based theories.

Other major perturbations were observed in the lactate shuttle for schizophrenia (Table 2) and major depressive disorder (Table 4). Lactate, a metabolic intermediate of glucose metabolism, also serves as an energetic substrate for the CNS. In the brain, lactate is transported between neurons and astrocytes via monocarboxylate transporters (MCTs) (Figure 1). Once in the cell, it may supply oxidative metabolism. The enzyme that limits the rate this pathway is lactate dehydrogenase (LDH) [68]. The availability of lactate is important for maintaining a functional synapse, and preclinical studies have reported cognitive impairment due to altered lactate substrate levels and brain pH in neuropsychiatric illnesses [69]. Our study, however, showed mixed findings regarding perturbations in this pathway, with various isozymes of the rate limiting enzymes being both upregulated and downregulated within the same disorder. Furthermore, the ketosis gene expression profile revealed only one perturbed gene, providing limited insight into whether ketone bodies modulate this pathway. Ultimately, our study confirms the perturbation of lactate metabolism in severe mental illnesses at the transcriptomic level, but further studies must assess whether ketones have any positive impact on this pathway at a therapeutic level. 

Fatty acids provide another major source of fuel for cells when carbohydrate metabolism is perturbed, or glucose and glycogen are scarce. Fatty acids may be obtained through the diet or synthesized endogenously. Fatty acid synthesis predominantly takes place in the cytoplasm of cells and is the process by which excess carbohydrates may be converted to fatty acids [70]. Fatty acid oxidation, also known as β-oxidation, is the process of utilizing fatty acids for fuel by catabolizing them to acetyl-CoA. In the process, the reducing agents NADH and flavin adenine dinucleotide (FADH_2_) are generated [71]. Like carbohydrate metabolism, fatty acid metabolism is also tightly hormonally regulated to maintain energy homeostasis and adapt to different nutritional conditions. While insulin promotes fatty acid synthesis by activating the enzymes acetyl-CoA carboxylase (ACC) and fatty acid synthase (FAS), glucagon inactivates these enzymes and promotes fatty acid oxidation. Additionally, the hormone leptin, acting on the hypothalamus, also modulates fatty acid metabolism by regulating food intake, controlling hunger, and monitoring overall energy expenditure [72]. Relative to the gene expression changes found in carbohydrate metabolism pathways, our study found fewer disruptions in fatty acid pathways (Table 2, Table 3 and Table 4). Based on the percentage of significant gene expression changes, fatty acid synthesis was perturbed in ketosis and bipolar disorder (Supplementary Tables S15 and 17); however, fatty acid metabolism was not among the top perturbed pathways based on the number of disrupted genes across any of the cohorts. In combination with the existing literature stating the numerous cognitive benefits of fatty acid metabolism in severe mental illnesses [73,74], our findings, overall, suggest that the modulation of fatty acid metabolism remains relatively intact, ensuring a stable energy supply in the face of metabolic challenges. However, additional studies on the hormonal and even transcriptional regulation of fatty acid metabolism will provide greater insights.

At the end of glycolysis or fatty acid oxidation, the metabolite acetyl-CoA faces one of two fates: it may either enter the TCA cycle for further metabolism or undergo ketogenesis to produce ketone bodies, both processes ultimately leading to ATP generation via oxidative phosphorylation [75]. The TCA cycle is localized within the matrix of mitochondria. Integrating many anabolic processes, such as gluconeogenesis and fatty acid synthesis, and catabolic processes that ultimately produce NADH, the TCA cycle is central to energy homeostasis. Carbohydrates, in the form of pyruvate, are converted to acetyl-CoA by the pyruvate dehydrogenase complex. The rate of this pathway is limited by citrate synthase (CS), where acetyl-CoA condenses with oxaloacetate to form citrate. The following catabolic steps produce the reducing equivalents NADH, FADH_2_, ATP, and carbon dioxide (CO_2_) [76]. Changes in TCA cycle enzymes and activity levels in neuropsychiatric illnesses have broadly been attributed to problems with energy homeostasis, mitochondrial integrity, neuroplasticity, and neurotransmission [7,49,77]. 

At the transcript level, our study confirmed significant perturbation of the TCA cycle across all three diseases. Schizophrenia showed an even ratio of upregulated to downregulated genes, with an approximately equal and small magnitude of expression change among genes; however, the rate-limiting enzyme CS was upregulated (Table 2). Similarly, major depressive disorder revealed predominantly upregulated genes with a greater magnitude of expression change compared to downregulated genes, and CS was also upregulated (Table 4). This may indicate an enhanced capacity for citrate production that ultimately leads to increased fatty acid synthesis. Given the glycolytic perturbations seen in schizophrenia and major depressive disorder in this study, a higher flux through the TCA cycle may be due to a need for alternate energy production in these disorders [47]. Bipolar disorder showed predominantly downregulated genes with a greater magnitude of expression change compared to upregulated genes (Table 3). CS was also downregulated, indicating a reduced capacity for citrate production, decreased substrate availability, and downregulation of the TCA cycle [78]. In combination with evidence for decreased glycolytic flux, this may indicate insufficient ATP generation via glucose-based metabolism and compromised cellular function in bipolar disorder.

An assessment of the expression of genes encoding enzymes in the TCA cycle across ketogenic datasets revealed significant downregulation of five genes: CS, IDH3G, SDHA, SDHB, and SUCLG2 (Table 2, Table 3 and Table 4). Comparing this expression profile to disease states revealed a largely discordant expression pattern with schizophrenia (Table 2) and major depressive disorder (Table 4). In both disease–ketosis comparisons, the discordant expression pattern with the rate-limiting enzyme CS aligns with the possibility that ketone bodies may correct pathogenic expression patterns to ensure a high bioenergetic output. Conversely, the bipolar–ketosis comparison of TCA cycle genes revealed a concordant expression pattern, with CS and IDH3G being significantly downregulated in both states (Table 3). This may indicate that ketone bodies stabilize a non-pathogenic process in which the TCA cycle may need to halt so that acetyl-CoA may be properly shunted towards ketogenesis, ultimately providing positive feedback to ketone generation for efficient energy production, mitochondrial integrity, and neurotransmission [75]. 

The alternative fate for acetyl-CoA is the production of ketone bodies via ketogenesis. Ketogenesis in a healthy brain, a state known as nutritional ketosis, typically occurs during prolonged fasting, exercise, or with the adoption of a ketogenic diet. During these conditions, heightened glucagon levels prompt an increase in carbohydrate metabolism to elevate blood glucose levels, resulting in increased gluconeogenesis, which ultimately depletes oxaloacetate (OAA), a critical intermediate of the TCA cycle. Once OAA is exhausted, the TCA cycle ceases, signaling a metabolic state akin to “starvation”, where all acetyl-CoA is redirected towards ketogenesis [79]. This leads to the production of ketone bodies such as acetoacetate, acetone, and beta-hydroxybutyrate. In the brain, ketone bodies are transported from astrocytes to neurons via MCTs. Within neurons, ketone bodies undergo ketolysis to regenerate acetyl-CoA, which may then re-enter the TCA cycle to produce ATP, circumventing glycolysis [22]. This process, ultimately, has numerous beneficial effects on the body and brain such as weight loss, improved blood sugar control, enhanced energy levels, and in the context of severe mental illnesses, neuroprotection, improved cognition, mood stabilization, and reduced inflammation [8,80,81,82]. Interestingly, exogenous ketone supplementation, either via ketone esters or ketone salts, also effectively increases blood ketone levels and provides many of the same benefits as a ketogenic diet in animal models of severe mental illness, without carbohydrate restriction [29,37].

Ketogenesis was not among the top significantly perturbed pathways across severe mental illnesses in our analysis based on a low number of gene expression changes (Table 2, Table 3 and Table 4). This observation aligns with the understanding that ketosis is not typically considered a pathogenic state in patients with these illnesses. Although not a top pathway based on the number of perturbed genes, ketogenesis was among the top disrupted pathways based on the percentage of perturbed genes in our schizophrenia and major depressive disorder studies (Supplementary Tables S16 and S18), with the magnitude of change being greater among the upregulated genes (LFC = 0.82 in schizophrenia, LFC = 0.62 in major depressive disorder) (Table 2) compared to the downregulated genes (LFC = −0.30 in schizophrenia, LFC = −0.35 in major depressive disorder) (Table 4), indicating, perhaps, a non-pathogenic metabolic rescue mechanism in the face of disrupted glucose consumption in these disease states. In addition, ketogenesis was not significantly perturbed in our ketogenic intervention study comprised of various ketogenic interventions compared to a standard diet. This may indicate that the ketogenic diet is effectively regulating the expression of these genes across different experimental conditions or animal models. It may also suggest that the metabolic pathways involved in ketogenesis are robust and resilient to perturbations in these specific contexts. However, these theories do not consider factors such as the duration of the ketogenic interventions, the models used in the studies, or the sensitivity of the methods employed to measure gene expression. Exploring other aspects of ketogenesis, such as metabolite levels or enzyme activity, will provide further insights into the metabolic effects of the ketogenic diet beyond the transcriptomic level.

The ETC is the final stage of cellular respiration, localized in the inner mitochondrial membrane, and is the major site for ATP production. The ETC comprises five protein complexes that utilize redox reactions to shuttle electrons from NADH to molecular oxygen (O_2_) with parallel proton transport across the inner mitochondrial membrane, creating an electrochemical gradient known as the mitochondrial membrane potential. This potential aids in the production of ATP generation via the ATP synthase (i.e., complex V) enzyme during chemiosmosis and oxidative phosphorylation. The rate of the ETC is limited by cytochrome C oxidase (COX), also known as complex IV, which catalyzes the final step of the ETC where electrons are transferred from cytochrome C to O_2_, resulting in the reduction from oxygen to water (H_2_O) [83,84]. 

The ETC showed the greatest number of gene expression changes across all three disorders (Table 2, Table 3 and Table 4) compared to control groups in our transcriptomic analysis, although this may have been attributed to the vast number of genes queried (*n* = 69) (Table 1). In schizophrenia, most of the perturbed genes were downregulated, including 10 isozymes encoding functional subunits of the rate-limiting enzyme COX (Table 2). However, the magnitude of expression change between schizophrenia and control groups across datasets among the downregulated genes was relatively small. Conversely, in major depressive disorder, most of the perturbed genes were upregulated, including nine isozymes that encode functional subunits of COX, with a moderate change in the expression of genes in disease compared to control groups across datasets (Table 4). Bipolar disorder showed an approximately even ratio of upregulated to downregulated genes with an approximately equal magnitude of expression change among genes (Table 3). Six isozymes of the rate-limiting enzyme were upregulated while three isozymes were downregulated, showing mixed results in terms of predominant upregulation or downregulation of this pathway in disease. These findings are in line with the current literature supporting altered ETC function and these perturbations being attributed to impaired oxidative stress, cellular resilience, inflammation, and disrupted mitochondrial dynamics that influence mood and cognitive impairments across neuropsychiatric illnesses [63,85].

In the ketogenic study, the majority of the perturbed ETC genes were downregulated, including three isozymes that transcribe functional units of COX [86] (Table 2, Table 3 and Table 4). In the schizophrenia–ketosis (Table 2) and bipolar–ketosis (Table 3) comparisons, a predominantly concordant expression was observed favoring downregulation of the ETC. In the major depressive disorder–ketosis comparison (Table 4), ETC genes showed a discordant expression pattern, with upregulation of the ETC in the disease and downregulation in the therapeutic state. The downregulation of the ETC genes in all three disease–ketosis comparisons may indicate that ketones offset a high amount of reactive oxygen species that are natural byproducts of electron transfer in the ETC [87,88]; however, further studies are needed to understand the implications of these transcriptomic changes.

The urea cycle, also known as the ornithine cycle, is another essential metabolic pathway for overall health and homeostasis. The urea cycle maintains nitrogen balance in the body and prevents the toxic buildup of ammonia, a byproduct of protein metabolism, by converting it into urea. This cycle primarily occurs in the liver but also has some activity in other tissues, including the brain [89]. In the urea cycle, ammonia is combined with CO_2_ and converted into urea through a series of enzymatic reactions. The rate of this pathway is limited by the enzyme carbamoyl phosphate synthetase I (CPSI), which catalyzes the formation of carbamoyl phosphate from ammonia and bicarbonate in the mitochondria. The remaining steps of this pathway occur in the cytosol to produce urea, which may be safely excreted by the kidneys [62]. Dysregulation of the urea cycle has been observed in some severe mental illnesses, such as bipolar disorder, where altered levels of urea cycle metabolites have been reported in the blood and brain tissue [90]. These findings suggest a potential link between disturbances in nitrogen metabolism and the pathophysiology of certain neuropsychiatric disorders. Unsurprisingly, in our study, the urea cycle was significantly perturbed based on the percentage of expression changes among genes in the bipolar disorder study (Table 3) as well as in schizophrenia (Table 2). The urea cycle was, overall, mildly perturbed based on the relatively low number of gene expression changes, with a notable downregulation of the rate-limiting enzyme in schizophrenia (Table 2) and major depressive disorder (Table 4), indicating a toxic buildup of metabolites. Further research is needed to understand the precise mechanisms underlying these associations and to explore the therapeutic potential of targeting the urea cycle in the treatment of severe mental illnesses.

Finally, considering that many of our datasets included data from individuals with neuropsychiatric illnesses who may have been taking medication, we examined the effect of antipsychotics and mood stabilizers on the expression of genes encoding metabolic enzymes in our core pathways of interest (Table 5). Since antipsychotics and mood stabilizers are typically prescribed chronically for schizophrenia and bipolar disorder, respectively (whereas antidepressants are often prescribed intermittently with the goal of tapering and discontinuation for major depressive disorder) [91,92], we focused on the effects of chronic medication. We analyzed transcriptomic studies where the expression of metabolic genes was measured only after subjects had been exposed to medication for at least 14 days. Our study found anywhere from *n* = 3 to *n* = 40 genes whose expression was altered across metabolic pathways based on chronic medication treatment. The most pronounced pathways, based on the highest number of gene expression changes, were the ETC, the TCA cycle, glycolysis, and fatty acid oxidation (Table 5). This differed from the top pathways affected by medication based on the highest percentage of genes with significant expression changes (Supplementary Tables S19 and S20). One limitation of this analysis is the use of animal model studies, which may have limited translational significance; however, our findings suggest that changes in metabolic gene expression may be secondary to chronic antipsychotic or mood stabilizer treatment rather than inherent to the disease process itself. These findings should be confirmed in a larger study using human or postmortem human tissue that considers the effect of different classes of antipsychotics, mood stabilizers, and non-psychotropic medications to determine the effect of medication on metabolic pathway enzyme-associated gene expression more precisely.

This study provides metabolic insights into neuropsychiatric illnesses and ketogenic therapies. Many of the reported results are consistent with the current literature, while other findings provide a foundation for further studies to fully understand the implications of metabolic changes at the transcriptomic level. A limitation of our analysis is the assortment of data stemming from RNA-Seq and microarray studies. These studies may not be directly comparable due to differences in techniques and sensitivities for detecting gene expression, which may have introduced inherent variability and noise in our results. Additionally, the limited number of available ketogenic intervention datasets may have impacted the robustness of our findings. As we continue to curate the ketosis module in Kaleidoscope with more data, our results are likely to become more comprehensive and reliable. Moreover, our analysis focused on 12 core metabolic pathways, overlooking other aspects of metabolism implicated in these disorders such as hormonal regulation [93,94,95], transcription factor regulation [96,97,98], mitochondrial dysfunction [62,63,99], ATP regulation via the purinergic system [100,101,102], and protein kinase regulation [103,104,105,106,107,108], which may provide a more comprehensive representation of bioenergetic dysregulation. Our study also did not account for sex differences due to the unavailability of these data in Kaleidoscope; however, considering sex differences in future studies is crucial, as the neuropsychiatric illnesses investigated show significant variations in prevalence and presentation between males and females [109,110]. Lastly, our study predominantly utilized brain-derived datasets, as we were interested in assessing brain bioenergetic dysfunction. For a more comprehensive understanding of systemic metabolic dysregulation across severe mental illnesses, integrating a wider array of datasets from various tissues and organs will be imperative in future work.

## 4. Materials and Methods

### 4.1. Filtering Metabolic Pathways and Genes of Interest 

Using the literature, major biochemical pathways involved in mammalian metabolism of macromolecules were identified and manually curated [111,112,113]. The gene annotation tool, Gene Ontology (GO) [114], was utilized to obtain the gene symbols associated with the major enzymes in each metabolic pathway. The Search Tool for the Retrieval of Interacting Genes/Proteins (STRING) database feature [115] within Kaleidoscope, an R shiny web application for in silico exploration of biological datasets [116], was subsequently utilized to obtain gene symbols for proteins known to interact with the enzymes of interest and refine the final gene sets of interest for each pathway. 

### 4.2. Building the “Ketosis” Module within the Kaleidoscope Lookup Tool

Kaleidoscope [116] contains a feature called “Lookup” that was utilized in our study for differential gene expression (DGE) analysis across published transcriptomic studies. Kaleidoscope allows end-users to process and upload user-defined DGE datasets; therefore, we built the “ketosis” module within the Lookup tool for our analysis. Our module contained eight RNA-Seq and microarray datasets derived from published animal model and cell culture studies that assessed the effects of ketogenic interventions between affected and control groups on different tissues, brain regions, and cell types. Of the eight studies, five were conducted with tissue or cells from the brain and three were conducted in tissue from the liver.

All datasets were curated using the National Center for Biotechnology Information Gene Expression Omnibus (NCBI GEO) genomics data repository [117,118]. Microarray datasets were processed utilizing the GEO2R analysis feature within GEO after defining case and control groups for each dataset. RNA-Seq datasets were processed utilizing Galaxy [119]. Briefly, for each RNA-Seq dataset, quality reports were generated using FastQC [120] and all quality reports were aggregated into a single report using MultiQC [121]. All reads were aligned to the appropriate reference genome using the HISAT2 alignment method [122]. Gene expression was measured on all aligned reads using FeatureCounts [123]. Case and control groups were defined, and the desired data were aggregated using Column Join. Finally, DGE analysis of the count data was performed using EdgeR [124]. The final DGE matrix for each ketogenic intervention dataset (*n* = 8) was uploaded into Kaleidoscope within the newly created “ketosis” module. Detailed information (e.g., species the study was conducted in, number of samples, source of tissue or cells, sequencing platform, raw dataset link for publicly available datasets, and associated manuscript) for each ketogenic transcriptomic dataset is provided in Supplementary Table S1. 

### 4.3. Querying Gene Lists and Generating Data Tables from Kaleidoscope Lookup

The gene list for each metabolic pathway in Table 1 was queried among the ketogenic datasets described as well as the disease datasets for neuropsychiatric illnesses of interest, modules for which already existed within the Kaleidoscope Lookup tab. A total of 35 schizophrenia, 55 bipolar disorder, and 36 major depressive disorder datasets were queried. These RNA-Seq and microarray datasets were derived from published human postmortem brain, animal, and cell culture model studies and processed in the same manner as the ketogenic studies. Detailed information (e.g., species the study was conducted in, number of samples, source of tissue or cells, sequencing platform, raw dataset link for publicly available datasets, and associated manuscript) for each disease-associated transcriptomic dataset is provided in Supplementary Tables S2–S4. 

Upon query, a data table containing log_2_ fold change (LFC) values and corresponding *p*-values for each gene and dataset within the specified disease– or ketogenic–pathway combination was retrieved and downloaded. The LFC value quantified the direction and magnitude of change in gene expression between two conditions being compared. A positive LFC value indicated that the gene’s expression level was higher in the experimental condition compared to the reference condition and a negative LFC value indicated that the gene’s expression level was lower in the experimental condition compared to the reference condition. The absolute value of the LFC indicated the magnitude of change in expression, where larger numbers represented more pronounced changes in gene expression between conditions. The *p*-value, after being corrected for multiple comparisons, indicated whether the gene was statistically significantly altered between the experimental and reference conditions. For 12 pathways and four modules, 48 total data tables, each containing the LFC and *p*-values associated with genes of interest across all datasets, were downloaded from Kaleidoscope for further processing. The workflow described thus far is displayed in Figure 2.

### 4.4. Transcriptomic Analysis among Neuropsychiatric Illness and Ketosis Datasets

Data tables were analyzed to assess the genes that survived correction for multiple comparisons and were significantly altered (*p* < 0.05) between experimental and control groups across available datasets within each module. For each disease–pathway and ketosis–pathway data table, each significant gene was identified, and its associated *p*-value was highlighted in yellow. The associated LFC value for the gene was highlighted in blue if the gene was significantly downregulated in the dataset being evaluated, or in red if the gene was significantly upregulated. For each gene, the number of datasets it was significantly upregulated in or downregulated in was summed independently. The average LFC for the significantly upregulated and downregulated genes was also calculated independently. A sample data table and the automation process used to conduct this analysis, as well as the Visual Basic code that was created in Excel Macro to automate this analysis, are provided in Supplementary Table S5, Supplementary Materials Methods, and the Supplementary Materials Appendix, respectively.

Next, for each disease–pathway or ketosis–pathway data table highlighted with significant data, a new data table was created that omitted non-significant data and contained only the following information: gene names, the number of datasets the gene was upregulated in, the average LFC among those datasets, the number of datasets the gene was downregulated in, and the average LFC among those datasets. For each gene, the number of datasets it was upregulated in was compared to the number of datasets it was downregulated in. Our advancement criteria threshold for considering a gene to be significantly dysregulated within a pathway was a ratio of two between these numbers (i.e., the gene was significantly upregulated or downregulated across at least two datasets within a module and the number of datasets the gene was significantly downregulated in was at least twice as many as it was upregulated in, or vice versa). The average LFC value among the datasets the gene was mostly dysregulated in was considered for further analysis. Any gene that did not have a ratio of at least two between upregulated to downregulated datasets, or vice versa, was not considered for further analysis. A sample data table displaying this information is provided in Supplementary Table S6. 

To determine the magnitude of change in expression across experimental vs. control groups among genes within a pathway, the LFC values for all significantly altered genes were further averaged. For each ketosis–pathway or disease–pathway comparison, the average, minimum, and maximum LFC values for significantly dysregulated genes are provided in Supplementary Tables S7–S10. Additionally, Supplementary Table S7 indicates whether the genes were altered in a ketogenic brain or liver study to differentiate brain and systemic metabolic changes. The automation process, as well as the Visual Basic code that was created in Excel Macro to conduct this analysis, is provided in the Supplementary Materials Methods and the Supplementary Materials Appendix, respectively.

The ketosis–pathway analysis was compared to each disease–pathway analysis (i.e., the ketosis–glycolysis analysis was compared to the schizophrenia–glycolysis analysis as well as to the bipolar–glycolysis and major depressive disorder–glycolysis analyses) to assess whether the genes that were significantly altered by pathway in the disease state were altered in the same (i.e., concordant) or opposite (i.e., discordant) direction in the ketogenic intervention datasets. For each disease–ketosis comparison, the names of the genes that were significantly dysregulated in each pathway, the direction of dysregulation, and the final average LFC value among the significantly altered genes (calculated in Supplementary Tables S7–S10) are presented in Table 2, Table 3 and Table 4. Information regarding the percentage of genes that were significantly altered in each pathway for ketosis, schizophrenia, bipolar disorder, and major depressive disorder are reported in Supplementary Tables S15–S18.

### 4.5. Analysis of the Effect of Antipsychotics and Mood Stabilizers on Metabolic Gene Expression

To understand the effect of medication on gene expression, differential metabolic gene expression was analyzed for all pathways across antipsychotic and mood stabilizer medication transcriptomic datasets in Kaleidoscope. The gene list for each metabolic pathway in Table 1 was queried among 24 chronic antipsychotic-treated vs. vehicle-treated (control) animal model datasets and seven chronic mood stabilizer-treated vs. vehicle-treated (control) animal model datasets. These RNA-Seq and microarray data were derived from published rodent model studies and processed (in the same manner as the ketogenic and neuropsychiatric illness studies) within the “antipsychotic” module in Kaleidoscope Lookup. Detailed information (e.g., drug name, drug dosage, drug administration period, species, treatment groups, brain region or cell type specificity, comparison groups in experiment, sequencing platform, raw dataset link for publicly available datasets, and associated manuscript) for each antipsychotic and mood stabilizer transcriptomic dataset is provided in Supplementary Table S11 and Supplementary Table S12, respectively.

Transcriptomic analysis was performed as previously described. Upon query, a data table containing LFC and corresponding *p*-values for each metabolic gene and dataset within the specified medication–pathway combination was retrieved and downloaded from Kaleidoscope for further processing. Data tables were analyzed to assess the genes that survived correction for multiple comparisons and were significantly altered (*p* < 0.05) between experimental and control groups. The same process used to derive Supplementary Tables S5–S10 was implemented. For each medication analysis (antipsychotics and mood stabilizers), the average, minimum, and maximum LFC values for significantly dysregulated genes grouped by metabolic pathway are provided in Supplementary Tables S13 and S14. The automation process as well as the Visual Basic code that was created in Excel Macro to conduct this analysis is provided in the Supplementary Materials Methods and the Supplementary Materials Appendix, respectively.

For each medication analysis, the genes that were significantly dysregulated in each pathway, the direction of dysregulation, and the final average LFC value among the significantly altered genes (calculated in Supplementary Tables S13 and S14) are presented in Table 5. Information regarding the percentage of genes that were significantly affected in each pathway by antipsychotics and mood stabilizers is reported in Supplementary Table S19 and Supplementary Table S20, respectively.

## 5. Conclusions

Metabolism is a dynamic process that involves a network of pathways finely tuned to maintain energy balance and support cellular activities. The observed metabolic perturbations from this study encompass numerous pathways, suggesting a multifaceted disruption of brain energy metabolism across neuropsychiatric illnesses. To reveal pathway dysregulation, our study uniquely scrutinized the directional changes of each gene—whether upregulated or downregulated—in experimental vs. control groups across several transcriptomic datasets. These findings provide a basis for understanding the heterogeneity of cell type etiology and behavioral phenotypes seen in severe mental illnesses. While these disorders may share genetic risk loci [125], those genetic variations manifest in complex ways due to bioenergetic systems that may be perturbed in a normal functioning brain. The brain bioenergetic gene expression profiles across schizophrenia, bipolar disorder, and major depressive disorder ultimately displayed dysfunctional pathways involving the catabolism of carbohydrates, suggesting inefficient generation of ATP from glucose: a disruption that necessitates alternative pathways for energy production to meet cellular energy demands. Given the far fewer perturbations of fatty acid metabolism, discordant ketogenic expression profiles in carbohydrate metabolism pathways across all disorders in this study, and prior evidence supporting the benefits of the ketogenic diet, further exploration into the therapeutic potential of ketogenic interventions in mitigating metabolic disruptions in neuropsychiatric disorders is warranted. Disparate data support the concept of brain bioenergetic dysfunction being a diverse feature of severe mental illnesses, contributing to the heterogeneity of phenotypes presented. Thus, future research should leverage the work presented to develop a global diagnostic tool capable of efficiently capturing metabolic profiles in psychiatric patients, thereby advancing our understanding and treatment of these challenging disorders.

## Figures and Tables

**Table 1:** Metabolic gene sets of interest organized by pathway.

Metabolic Pathway (*n* = 12)	Rate-Limiting Enzyme	Final Gene List (Output from Kaleidoscope STRING)	Number of Genes (*n* = 242)
Gluconeogenesis	Fructose-1,6- bisphosphate	PC, PCK1, PCK2, **FBP1**, FBP2, G6PC1, G6PC2, G6PC3, PFKFB2	9
Glycolysis	Phosphofructokinase	HK1, HK2, HK3, GCK, GPI, **PFKL**, **PFKM**, **PFKP**, ALDOA, ALDOB, ALDOC, TPI1, GAPDH, GAPDHS, PGK1, PGK2, BPGM, PGAM1, PGAM2, PGAM4, ENO1, ENO2, ENO3, ENO4, PKLR, PKM	27
Lactate Shuttle (Neuron–Astrocyte)	Lactate dehydrogenase	SLC2A3, **LDHA**, LDHC, LDHD, SLC16A1, SLC16A3, GLS, GLS2, GLUL, SLC2A1, SLC2A10, SLC2A11, SLC2A12, SLC2A14, SLC1A1, SLC1A2, SLC1A3, SLC1A6	18
Tricarboxylic Acid (TCA) Cycle	Citrate synthase	**CS**, ACLY, ACO1, ACO2, IREB2, IDH1, IDH2, IDH3A, IDH3B, IDH3G, OGDH, OGDHL, SUCLA2, SUCLG2, SUCLG1, SDHA, SDHB, SDHC, SDHD, SDHAF1, SDHAF4, ALDH5A1, FH, MDH1B, MDH2, PDHA1, PDHA2, DBT, DLAT, DLD	30
Electron Transport Chain (ETC)	Cytochrome C oxidase	NDUFA8, NDUFS4, NDUFV3, NDUFA11, NDUFS5, NDUFC1, NDUFC2, NDUFS1, NDUFV2, NDUFV1, NDUFA12, NDUFB5, MT-ND1, MT-ND2, MT-ND3, MT-ND4, MT-ND5, MT-ND6, MT-ND4L, SDHA, SDHB, SDHC, SDHD, SDHAF1, SDHAF2, SDHAF3, SDHAF4, UQCRB, UQCRQ, UQCRC1, UQCRC2, UQCR10, MT-CYB, CYC1, UQCRFS1, UQCRH, UQCR10, UQCR11, **COX4I1**, **COX4I2**, **COX5A**, **COX5B**, **COX6A1**, **COX6A2**, **COX6B1**, **COX6B2**, **COX6C**, **COX7A1**, **COX7A2**, **COX7B**, **COX7B2**, **COX7C**, **COX8A**, **COX8C**, **MT-CO1**, **MT-CO2**, **MT-CO3**, COQ4, COQ7, COQ10A, COQ10B, CYCS, ATP5F1A, ATP5F1B, ATP5F1C, ATP5F1D, ATP5F1E, MT-ATP6, MT-ATP8	69
Fatty Acid Synthesis	Acetyl-CoA carboxylase	**ACACA**, ACACB, MCAT, FASN, OXSM, DECR1, HADH, ELOVL1, ELOVL3, ELOVL6, OLAH, PPT1, PPT2	13
Fatty Acid Oxidation	Carnitine palmitoyltransferase I	SLC27A2, ACSBG2, ACSBG1, ECI1, ECHS1, HADHA, HSD17B10, HADH, HADHB, ACAA2, SCP2, VLCAD, SCAD, MCAD, LCAD, **CPT1C**, CPT2	17
Ketogenesis	HMG-CoA synthase	ACAT1, ACAA1, ACAA2, **HMGCS1**, HMGCS2, HMGCL, HMGCLL1, BDH2, BDH1	9
Glycogenesis	Glycogen synthase	ADPGK, GCKR, HK1, HK2, HK3, PGM1, PGM2, UGP2, **GYS1**, **GYS2**, GSK3A, GSK3B, GBE1	13
Glycogenolysis	Glycogen phosphorylase	**PYGL**, **PYGM**, PYGB, AGL, PGM1, PGM2, G6PC1, G6PC2, G6PC3	9
Urea Cycle	Carbamoyl phosphate synthetase I	OTC, OAT, **CPS1**, CAD, ASS1, ASL, ARG1, ARG2, AGMAT, NAGS, ACY1	11
Pentose Phosphate/ Glutathione Pathways	Glucose-6-phosphate dehydrogenase	HK1, HK2, HK3, **G6PD**, PGD, TKT, TKTL1, TKTL2, TALDO1, GPI, GCLC, GSS, GSR, SOD1, RPE, RPIA, RGN	17

The rate-limiting enzyme for each metabolic pathway is listed in column two, and the gene(s) that encode these enzymes are bolded in the gene list in column three. Gene lists indicate the genes that were investigated in the Kaleidoscope Lookup study to assess dysregulation at the mRNA level within each pathway (column three). The number of genes in each pathway of interest is stated concisely in column four.

## Data Availability

Data are contained within the Article and Supplementary Materials.

## Supplementary Materials

The following supporting information can be downloaded at https://www.mdpi.com/article/10.3390/ijms25158266/s1.
