# Supplementary material for: Metabolic Insights into Neuropsychiatric Illnesses and Ketogenic Therapies: A Transcriptomic View
Source: Int J Mol Sci. 2024 Jul 29;25(15):8266. doi: 10.3390/ijms25158266 (PMC11312282; doi:10.3390/ijms25158266)
Supplement: Supplementary file 1 [file ijms-25-08266-s001.zip › Supplementary Materials.pdf]

Metabolic Insights into Neuropsychiatric Illnesses and Ketogenic Therapies: A Transcriptomic View| Supplementary Materials

Contents	
Supplementary Methods	Page 2
Supplementary Tables – Titles & Captions Only	Page 3
Supplementary Tables with Titles & Captions	Page 7
Appendix	Page 13

Supplementary Methods: Automation of data analysis:

The transcriptomic analysis that was conducted to differentiate the significant differentially expressed genes and their associated $\log_2$-fold change (LFC) values among schizophrenia, bipolar disorder, major depressive disorder, and ketogenic intervention datasets was automated utilizing Visual Basic code in Excel Macro. The analysis conducted for each disease-pathway and ketogenic intervention-pathway displaying significant differentially expressed genes, direction of dysregulation (i.e., upregulated or downregulated), number of datasets the gene was dysregulated in, and average LFC values among those datasets for each gene was automated utilizing Visual Basic code in Excel Macro. Finally, the medication (antipsychotic and mood stabilizer) analysis was also automated utilizing Visual Basic code in Excel Macro. The written code with comments for all processes described may be seen in the **Appendix** section of this document.

The automation process in all analyses involved the following steps: Raw Excel files were downloaded. The "Developer" tab was selected followed by the "Visual Basic" button, which opened a new window. "Insert Module" was selected, which opened a platform in which to write the code. Next, in Excel, "Developer" was again selected followed by "Macros". The appropriate code was selected and run on the data of interest.

Supplementary Tables – Titles & Captions:

Note: All supplementary tables are provided in the "Supplementary Tables" excel file. Only the titles and captions are provided below.

Supplementary Table 1: Ketosis datasets in the Kaleidoscope Lookup tool. For each dataset within the ketosis module (n=8), information regarding the species the study was conducted in, details on how the experiments were conducted, the number of samples (cases vs. controls), the source of tissue or cells, brain region or cell level specificity (if applicable), the sequencing platform, a link to the raw dataset for publicly available datasets, and the associated manuscript, if available, is provided.

Supplementary Table 2: Schizophrenia datasets in the Kaleidoscope Lookup tool. For each dataset of interest within the schizophrenia module (n=35), information regarding the species the study was conducted in, details on how the experiments were conducted, the number of samples (cases vs. controls), the source of tissue or cells, brain region or cell level specificity, the sequencing platform, a link to the raw dataset for publicly available datasets, and the associated manuscript, if available, is provided.

Supplementary Table 3: Bipolar disorder datasets in the Kaleidoscope Lookup tool. For each dataset of interest within the bipolar disorder module (n=55), information regarding the species the study was conducted in, details on how the experiments were conducted, the number of samples (cases vs. controls), the source of tissue or cells, brain region or cell level specificity, the sequencing platform, a link to the raw dataset for publicly available datasets, and the associated manuscript, if available, is provided.

Supplementary Table 4: Major depressive disorder datasets in the Kaleidoscope Lookup tool. For each dataset of interest within the major depressive disorder module (n=36), information regarding the species the study was conducted in, details on how the experiments were conducted, the number of samples (cases vs. controls), the source of tissue or cells, brain region or cell level specificity, the sequencing platform, a link to the raw dataset for publicly available datasets, and the associated manuscript, if available, is provided.

Supplementary Table 5: Transcriptomic analysis sample data table. Sample data table for the glycolysis pathway in schizophrenia. Columns indicate the datasets in the schizophrenia module within Kaleidoscope Lookup and rows indicate all genes of interest in the glycolysis pathway (i.e., genes from Table 1 that were queried). The data in the table shows the p-value and $\log_2$-fold change (LFC) value for each gene in each dataset. The visual basic code was applied to highlight the p-values of all significant genes

that survived correction for multiple comparisons in each dataset in yellow ($p<0.05$). If the associated LFC value was positive (upregulated), the corresponding cell was highlighted in red. If the associated LFC value was negative (downregulated) the corresponding cell was highlighted in blue. In columns 'BT' and 'BU' in the table titled "Up Regulated" and "Down Regulated," the number of times each gene was upregulated and downregulated across all datasets was summed, respectively. In columns 'BV' and 'BW' in the excel sheet titled "Up Regulated LFC Avg" and "Down Regulated LFC Avg," the associated LFC values for significantly upregulated and downregulated genes was averaged, respectively. This analysis was done for all 12 pathways in each disease (n=3) and ketosis state.

Supplementary Table 6: Concise transcriptomic analysis sample data table. Concise sample data table for the glycolysis pathway in schizophrenia. Non-significant data and individual dataset information from Supplementary Table 5 was omitted. The gene names, number of datasets each gene was upregulated and downregulated in, and average LFC values for significant datasets was carried forward. This concise data table was utilized to assess whether each gene was upregulated or downregulated across majority of the datasets (see section *2.4. Transcriptomic analysis among neuropsychiatric illness and ketosis datasets* in Methods). If the gene was upregulated across majority of the datasets, the first and third column values for that gene was highlighted in red. If the gene was downregulated across majority of the datasets, the second and fourth column values for that gene was highlighted in blue. The final pathway LFC value was calculated as the average of the highlighted LFC values for upregulated and downregulated genes independently. Final LFC values are bolded in the table. These are the same values displayed in **Tables 2-4** in the body of the manuscript.

Supplementary Table 7: Final pathway analysis tables for datasets in ketosis. 11 independent tables for each pathway showing significant genes, whether they're upregulated or downregulated, how many datasets they're upregulated or downregulated in, whether the dataset is from a study conducted in the brain or liver, each gene's average LFC value across datasets, final LFC values among upregulated and downregulated genes independently, and the range of average LFC values (min and max values). A table for the "ketogenesis" pathway is not shown since no genes were significantly altered based on the threshold criteria outlined in the Methods section for this comparison.

Supplementary Table 8: Final pathway analysis tables for datasets in schizophrenia. 12 independent tables for each pathway showing significant genes, whether they're upregulated or downregulated, how many datasets they're upregulated or downregulated in, each gene's average LFC value across datasets, final LFC values

among upregulated and downregulated genes independently, and the range of average LFC values (min and max values).

Supplementary Table 9: Final pathway analysis tables for datasets in bipolar disorder. 12 independent tables for each pathway showing significant genes, whether they're upregulated or downregulated, how many datasets they're upregulated or downregulated in, each gene's average LFC value across datasets, final LFC values among upregulated and downregulated genes independently, and the range of average LFC values (min and max values).

Supplementary Table 10: Final pathway analysis tables for datasets in major depressive disorder. 12 independent tables for each pathway showing significant genes, whether they're upregulated or downregulated, how many datasets they're upregulated or downregulated in, each gene's average LFC value across datasets, final LFC values among upregulated and downregulated genes independently, and the range of average LFC values (min and max values).

Supplementary Table 11: Antipsychotic datasets in the Kaleidoscope Lookup tool. For each dataset within the ketosis module (n=24), information regarding the drug name, drug class, dosage given in the experiment, chronic drug administration period (chronic defined by a period of at least two weeks), species the study was conducted in, brain region, treatment groups, brain region or cell level specificity, disease or treatment comparison, sequencing platform, a link to the raw dataset for publicly available datasets, and associated manuscript, if available, is provided.

Supplementary Table 12: Mood stabilizer datasets in the Kaleidoscope Lookup tool. For each dataset within the ketosis module (n=7), information regarding the drug name, drug class, dosage given in the experiment, chronic drug administration period (chronic defined by a period of at least two weeks), species the study was conducted in, brain region, treatment groups, brain region or cell level specificity, disease or treatment comparison, sequencing platform, a link to the raw dataset for publicly available datasets, and associated manuscript, if available, is provided.

Supplementary Table 13: Significantly altered genes between antipsychotic treatment vs. control groups by pathway. 12 independent tables for each pathway showing significant genes, whether they're upregulated or downregulated, how many datasets they're upregulated or downregulated in, each gene's average LFC value across datasets, final LFC values among upregulated and downregulated genes independently, and the range of average LFC values (min and max values).

Supplementary Table 14: Significantly altered genes between mood stabilizer treatment vs. control groups by pathway. 12 independent tables for each pathway showing significant genes, whether they're upregulated or downregulated, how many datasets they're upregulated or downregulated in, each gene's average LFC value across datasets, final LFC values among upregulated and downregulated genes independently, and the range of average LFC values (min and max values).

Supplementary Table 15: Differential gene expression analysis by pathway for ketosis.

KETOSIS	
	**Overall (N=242)**
**Gluconeogenesis (n=9)**	**Fatty Acid Oxidation (n=17)**
Upregulated: 11% (n=1)	Upregulated: 6% (n=1)
Downregulated: 11% (n=1)	Downregulated: 0% (n=0)
Overall: **22%** (n=2)	Overall: 6% (n=1)
**Glycolysis (n=27)**	**Ketogenesis (n=9)**
Upregulated: 0% (n=0)	Upregulated: 0% (n=0)
Downregulated: 11% (n=3)	Downregulated: 0% (n=0)
Overall: 11% (n=3)	Overall: 0% (n=0)
**Lactate Shuttle (Neuron-Astrocyte) (n=18)**	**Glycogenesis (n=13)**
Upregulated: 6% (n=1)	Upregulated: 0% (n=0)
Downregulated: 0% (n=0)	Downregulated: 8% (n=1)
Overall: 6% (n=1)	Overall: 8% (n=1)
**Tricarboxylic Acid (TCA) Cycle (n=30)**	**Glycogenolysis (n=9)**
Upregulated: 0% (n=0)	Upregulated: 22% (n=2)
Downregulated: 17% (n=5)	Downregulated: 11% (n=1)
Overall: 17% (n=5)	Overall: **33%** (n=3)
**Electron Transport Chain (ETC) (n=69)**	**Urea Cycle (n=11)**
Upregulated: 4% (n=3)	Upregulated: 0% (n=0)
Downregulated: 16% (n=11)	Downregulated: 9% (n=1)
Overall: 20% (n=14)	Overall: 9% (n=1)
**Fatty Acid Synthesis (n=13)**	**Pentose Phosphate/Glutathione (n=17)**
Upregulated: 15% (n=2)	Upregulated: 0% (n=0)
Downregulated: 15% (n=2)	Downregulated: 18% (n=3)
Overall: **30%** (n=4)	Overall: 18% (n=3)

Table showing the percent and number of genes that were significantly dysregulated ($p<0.05$) and survived correction for multiple comparisons across ketosis datasets (n=8) in our Kaleidoscope "Lookup" study. Highlighted cells indicate the top three dysregulated pathways based on the highest percent of altered genes: Glycogenolysis (33%), Fatty Acid Synthesis (30%), and Gluconeogenesis (22%). The top three dysregulated pathways based on the highest number of altered genes were the Electron Transport Chain (ETC) (n=14), Tricarboxylic Acid (TCA) Cycle (n=5), and Fatty Acid Synthesis (n=4).

Supplementary Table 16: Differential gene expression analysis by pathway for schizophrenia.

SCHIZOPHRENIA	
	**Overall (N=242)**
**Gluconeogenesis (n=9)**	**Fatty Acid Oxidation (n=17)**
Upregulated: 22% (n=2)	Upregulated: 29% (n=5)
Downregulated: 22% (n=2)	Downregulated: 6% (n=1)
Overall: 44% (n=4)	Overall: 35% (n=6)
**Glycolysis (n=27)**	**Ketogenesis (n=9)**
Upregulated: 33% (n=9)	Upregulated: 44% (n=4)
Downregulated: 19% (n=5)	Downregulated: 22% (n=2)
Overall: 52% (n=14)	Overall: **66%** (n=6)
**Lactate Shuttle (Neuron-Astrocyte) (n=18)**	**Glycogenesis (n=13)**
Upregulated: 33% (n=6)	Upregulated: 8% (n=1)
Downregulated: 28% (n=5)	Downregulated: 31% (n=4)
Overall: 61% (n=11)	Overall: 39% (n=5)
**Tricarboxylic Acid (TCA) Cycle (n=30)**	**Glycogenolysis (n=9)**
Upregulated: 23% (n=7)	Upregulated: 11% (n=1)
Downregulated: 23% (n=7)	Downregulated: 11% (n=1)
Overall: 46% (n=14)	Overall: 22% (n=2)
**Electron Transport Chain (ETC) (n=69)**	**Urea Cycle (n=11)**
Upregulated: 6% (n=4)	Upregulated: 27% (n=3)
Downregulated: 51% (n=35)	Downregulated: 36% (n=4)
Overall: 57% (n=39)	Overall: **63%** (n=7)
**Fatty Acid Synthesis (n=13)**	**Pentose Phosphate/Glutathione (n=17)**
Upregulated: 31% (n=4)	Upregulated: 29% (n=5)
Downregulated: 31% (n=4)	Downregulated: 29% (n=5)
Overall: **62%** (n=8)	Overall: 58% (n=10)

Table showing the percent and number of genes that were significantly dysregulated ($p<0.05$) and survived correction for multiple comparisons across schizophrenia datasets (n=35) in our Kaleidoscope "Lookup" study. Highlighted cells indicate the top three dysregulated pathways based on the highest percent of altered genes: Ketogenesis (66%), Urea Cycle (63%), and Fatty Acid Synthesis (62%). The top three dysregulated pathways based on the highest number of altered genes were the Electron Transport Chain (ETC) (n=39), Tricarboxylic Acid (TCA) Cycle (n=14), and Lactate Shuttle (Neuron-Astrocyte) (n=11).

Supplementary Table 17: Differential gene expression analysis by pathway for bipolar disorder.

BIPOLAR DISORDER	
	**Overall (N=242)**
**Gluconeogenesis (n=9)**	**Fatty Acid Oxidation (n=17)**
Upregulated: 22% (n=2)	Upregulated: 18% (n=3)
Downregulated: 11% (n=1)	Downregulated: 18% (n=3)
Overall: 33% (n=4)	Overall: 36% (n=6)
**Glycolysis (n=27)**	**Ketogenesis (n=9)**
Upregulated: 15% (n=4)	Upregulated: 22% (n=2)
Downregulated: 44% (n=12)	Downregulated: 33% (n=3)
Overall: 59% (n=16)	Overall: 55% (n=5)
**Lactate Shuttle (Neuron-Astrocyte) (n=18)**	**Glycogenesis (n=13)**
Upregulated: 28% (n=5)	Upregulated: 23% (n=3)
Downregulated: 17% (n=3)	Downregulated: 8% (n=1)
Overall: 45% (n=8)	Overall: 31% (n=4)
**Tricarboxylic Acid (TCA) Cycle (n=30)**	**Glycogenolysis (n=9)**
Upregulated: 10% (n=3)	Upregulated: 11% (n=1)
Downregulated: 40% (n=12)	Downregulated: 33% (n=3)
Overall: 50% (n=15)	Overall: 44% (n=4)
**Electron Transport Chain (ETC) (n=69)**	**Urea Cycle (n=11)**
Upregulated: 28% (n=19)	Upregulated: 27% (n=3)
Downregulated: 35% (n=24)	Downregulated: 45% (n=5)
Overall: **63%** (n=43)	Overall: **72%** (n=8)
**Fatty Acid Synthesis (n=13)**	**Pentose Phosphate/Glutathione (n=17)**
Upregulated: 31% (n=4)	Upregulated: 24% (n=4)
Downregulated: 38% (n=5)	Downregulated: 24% (n=4)
Overall: **69%** (n=9)	Overall: 48% (n=8)

Table showing the percent and number of genes that were significantly dysregulated ($p<0.05$) and survived correction for multiple comparisons across bipolar disorder datasets (n=55) in our Kaleidoscope "Lookup" study. Highlighted cells indicate the top three dysregulated pathways based on the highest percent of altered genes: Urea Cycle (72%), Fatty Acid Synthesis (69%), and the Electron Transport Chain (ETC) (63%). The top three dysregulated pathways based on the highest number of altered genes were the Electron Transport Chain (ETC) (n=43), Glycolysis (n=16), and Tricarboxylic Acid (TCA) Cycle (n=15).

Supplementary Table 18: Differential gene expression analysis by pathway for major depressive disorder.

MAJOR DEPRESSIVE DISORDER	
	**Overall (N=242)**
**Gluconeogenesis (n=9)**	**Fatty Acid Oxidation (n=17)**
Upregulated: 11% (n=1)	Upregulated: 17% (n=3)
Downregulated: 33% (n=3)	Downregulated: 24% (n=4)
Overall: 44% (n=4)	Overall: 41% (n=7)
**Glycolysis (n=27)**	**Ketogenesis (n=9)**
Upregulated: 44% (n=12)	Upregulated: 22% (n=2)
Downregulated: 7% (n=2)	Downregulated: 55% (n=5)
Overall: 51% (n=14)	Overall: **77%** (n=7)
**Lactate Shuttle (Neuron-Astrocyte) (n=18)**	**Glycogenesis (n=13)**
Upregulated: 22% (n=4)	Upregulated: 23% (n=3)
Downregulated: 44% (n=8)	Downregulated: 23% (n=3)
Overall: 66% (n=12)	Overall: 46% (n=6)
**Tricarboxylic Acid (TCA) Cycle (n=30)**	**Glycogenolysis (n=9)**
Upregulated: 50% (n=15)	Upregulated: 22% (n=2)
Downregulated: 27% (n=8)	Downregulated: 55% (n=5)
Overall: **77%** (n=23)	Overall: **77%** (n=7)
**Electron Transport Chain (ETC) (n=69)**	**Urea Cycle (n=11)**
Upregulated: 51% (n=35)	Upregulated: 27% (n=3)
Downregulated: 7% (n=5)	Downregulated: 27% (n=3)
Overall: 58% (n=40)	Overall: 54% (n=6)
**Fatty Acid Synthesis (n=13)**	**Pentose Phosphate/Glutathione (n=17)**
Upregulated: 23% (n=3)	Upregulated: 53% (n=9)
Downregulated: 23% (n=3)	Downregulated: 0% (n=0)
Overall: 46% (n=6)	Overall: 53% (n=9)

Table showing the percent and number of genes that were significantly dysregulated ($p<0.05$) and survived correction for multiple comparisons across major depressive disorder datasets (n=36) in our Kaleidoscope "Lookup" study. Highlighted cells indicate the top three dysregulated pathways based on the highest percent of altered genes: Tricarboxylic Acid (TCA) Cycle (77%), Ketogenesis (77%), and Glycogenolysis (77%). The top three dysregulated pathways based on the highest number of altered genes were the Electron Transport Chain (ETC) (n=40), Tricarboxylic Acid (TCA) Cycle (n=23), and Glycolysis (n=14).

Supplementary Table 19: Effect of chronic antipsychotics on metabolic differential gene expression.

ANTIPSYCHOTICS	
	**Overall (N=242)**
**Gluconeogenesis (n=9)**	**Fatty Acid Oxidation (n=17)**
Upregulated: 33% (n=3)	Upregulated: 29% (n=5)
Downregulated: % (n=0)	Downregulated: 0% (n=0)
Overall: 33% (n=3)	Overall: 29% (n=5)
**Glycolysis (n=27)**	**Ketogenesis (n=9)**
Upregulated: 22% (n=6)	Upregulated: 44% (n=4)
Downregulated: 15% (n=4)	Downregulated: 0% (n=0)
Overall: 37% (n=10)	Overall: 44% (n=4)
**Lactate Shuttle (Neuron-Astrocyte) (n=18)**	**Glycogenesis (n=13)**
Upregulated: 22% (n=4)	Upregulated: 15% (n=2)
Downregulated: 28% (n=5)	Downregulated: 38% (n=5)
Overall: 50% (n=9)	Overall: **53%** (n=7)
**Tricarboxylic Acid (TCA) Cycle (n=30)**	**Glycogenolysis (n=9)**
Upregulated: 17% (n=5)	Upregulated: 11% (n=1)
Downregulated: 17% (n=5)	Downregulated: 33% (n=3)
Overall: 34% (n=10)	Overall: 44% (n=4)
**Electron Transport Chain (ETC) (n=69)**	**Urea Cycle (n=11)**
Upregulated: 16% (n=11)	Upregulated: 27% (n=3)
Downregulated:10 % (n=7)	Downregulated: 9% (n=1)
Overall: 26% (n=18)	Overall: 36% (n=4)
**Fatty Acid Synthesis (n=13)**	**Pentose Phosphate/Glutathione (n=17)**
Upregulated: 31% (n=4)	Upregulated: 41% (n=7)
Downregulated: 31% (n=4)	Downregulated: 12% (n=2)
Overall: **62%** (n=8)	Overall: **53%** (n=9)

Table showing the percent and number of genes that were significantly dysregulated ($p<0.05$) and survived correction for multiple comparisons across chronic antipsychotic datasets (n=24) in our Kaleidoscope "Lookup" study. Highlighted cells indicate the top three dysregulated pathways based on the highest percent of altered genes: Fatty Acid Synthesis (62%), Glycogenesis (53%), and Pentose Phosphate/Glutathione Pathways (53%). The top three dysregulated pathways based on the highest number of altered genes were the Electron Transport Chain (ETC) (n=18), Tricarboxylic Acid (TCA) Cycle (n=10), and Glycolysis (n=10).

Supplementary Table 20: Effect of chronic mood stabilizers on metabolic differential gene expression.

MOOD STABILIZERS	
	**Overall (N=242)**
**Gluconeogenesis (n=9)**	**Fatty Acid Oxidation (n=17)**
Upregulated: 0% (n=0)	Upregulated: 35% (n=6)
Downregulated: 33% (n=3)	Downregulated: 0% (n=0)
Overall: **33%** (n=3)	Overall: **35%** (n=6)
**Glycolysis (n=27)**	**Ketogenesis (n=9)**
Upregulated: 4% (n=1)	Upregulated: 0% (n=0)
Downregulated: 7% (n=2)	Downregulated: 33% (n=3)
Overall: 11% (n=3)	Overall: **33%** (n=3)
**Lactate Shuttle (Neuron-Astrocyte) (n=18)**	**Glycogenesis (n=13)**
Upregulated: 11% (n=2)	Upregulated: 23% (n=3)
Downregulated: 11% (n=2)	Downregulated: 8% (n=1)
Overall: 22% (n=4)	Overall: 31% (n=4)
**Tricarboxylic Acid (TCA) Cycle (n=30)**	**Glycogenolysis (n=9)**
Upregulated: 13% (n=4)	Upregulated: 22% (n=2)
Downregulated: 10% (n=3)	Downregulated: 0% (n=0)
Overall: 23% (n=7)	Overall: 22% (n=2)
**Electron Transport Chain (ETC) (n=69)**	**Urea Cycle (n=11)**
Upregulated: 7% (n=5)	Upregulated: 9% (n=1)
Downregulated: 3% (n=2)	Downregulated: 9% (n=1)
Overall: 10% (n=7)	Overall: 18% (n=2)
**Fatty Acid Synthesis (n=13)**	**Pentose Phosphate/Glutathione (n=17)**
Upregulated: 8% (n=1)	Upregulated: 0% (n=0)
Downregulated: 0% (n=0)	Downregulated: 12% (n=2)
Overall: 8% (n=1)	Overall: 12% (n=2)

Table showing the percent and number of genes that were significantly dysregulated ($p<0.05$) and survived correction for multiple comparisons across chronic mood stabilizer datasets (n=7) in our Kaleidoscope "Lookup" study. Highlighted cells indicate the top three dysregulated pathways based on the highest percent of altered genes: Fatty Acid Oxidation (35%), Gluconeogenesis (33%), and Ketogenesis (33%). The top three dysregulated pathways based on the highest number of altered genes were the Electron Transport Chain (ETC) (n=7), Tricarboxylic Acid (TCA) Cycle (n=7), and Fatty Acid Oxidation (n=6).

Appendix: Code for the automation process described in the **Supplementary Methods**:

*The output data table after the application of this code may be seen in **Supplementary Table 5**:*

```
Sub findSigGenesAndLFCValues()
   Dim lastColumn As Long
   Dim lastRow As Long
   Dim currentColumn As Long

   'Insert a row between rows 1 and 2
   Rows(2).Insert Shift:=xlDown

   'Find the last column with data in the first row
   lastColumn = Cells(1, Columns.Count).End(xlToLeft).Column

   'Loop through columns and add new columns
   For currentColumn = 3 To lastColumn * 2 Step 2
      Columns(currentColumn).Insert Shift:=xlToRight,
CopyOrigin:=xlFormatFromLeftOrAbove

   Next currentColumn

   'Find the last column with data in the first row
   lastColumn = Cells(1, Columns.Count).End(xlToLeft).Column

   'Loop through columns
   For currentColumn = 2 To lastColumn Step 2
      ' Find the last row with data in the current column
      lastRow = Cells(Rows.Count, currentColumn).End(xlUp).Row

      'Split values by comma
      Range(Cells(2, currentColumn), Cells(lastRow, currentColumn)).TextToColumns
Destination:=Range(Cells(2, currentColumn), Cells(lastRow, currentColumn)),
DataType:=xlDelimited, _
         TextQualifier:=xlDoubleQuote, ConsecutiveDelimiter:=False, Tab:=False, _
         Semicolon:=False, Comma:=True, Space:=False, Other:=False, FieldInfo _
         :=Array(1, 1), TrailingMinusNumbers:=True

      'Find and replace "p=" with ""
      Columns(currentColumn + 1).Replace "p=", "", xlPart, xlByColumns
```

```
        'Add col headers
        Cells(2, currentColumn).Value = "LFC"
        Cells(2, currentColumn + 1).Value = "p Value"

        'Change blank cells to "NA"
        ChangeBlankCellsToNA Range(Cells(2, currentColumn + 1), Cells(lastRow,
currentColumn + 1))

        'Highlight p values less that 0.05
        Dim rng As Range
        Set rng = Range(Cells(2, currentColumn + 1), Cells(lastRow, currentColumn + 1))
        HighlightValuesInRange rng, 0, 0.05
    Next currentColumn

    'Write column names of last columns
    Cells(2, currentColumn).Value = "Up Regulated"
    Cells(2, currentColumn + 1).Value = "Down Regulated"
    Cells(2, currentColumn + 2).Value = "Up Regulated LFC Avg"
    Cells(2, currentColumn + 3).Value = "Down Regulated LFC Avg"

    'Color up or down regulated cells
    LFC_Color_Up_Down
End Sub

'Highlight significant p values
Sub HighlightValuesInRange(rng As Range, minValue As Double, maxValue As Double)
    Dim cell As Range

    For Each cell In rng
        If cell.Value >= minValue And cell.Value < maxValue Then
            cell.Interior.Color = RGB(255, 255, 0) ' Change the color as needed
        End If
    Next cell

End Sub

'Change empty cells to "NA"
Sub ChangeBlankCellsToNA(rng As Range)
    Dim cell As Range
```

```
    For Each cell In rng
        If IsEmpty(cell.Value) Then
            cell.Value = "NA"
        End If
    Next cell
End Sub

'Color cells that are up or down regulated
Sub LFC_Color_Up_Down()
    'define variables
    Dim currentColumn As Long
    Dim lastColumn As Long
    Dim lastRow As Long
    Dim currentRow As Long
    Dim upRegCount As Long
    Dim downRegCount As Long

    'Initialize variables
    lastColumn = Cells(1, Columns.Count).End(xlToLeft).Column
    lastRow = Cells(Rows.Count, 1).End(xlUp).Row
    upRegCount = 0
    downRegCount = 0
    upRegTot = 0
    downRegTot = 0

    'Loop through data starting with row 3
    For currentRow = 3 To lastRow 'Assuming your data starts from row 3
        For currentColumn = 2 To lastColumn Step 2
            If Cells(currentRow, currentColumn + 1).Value < 0.05 Then
                If Cells(currentRow, currentColumn).Value > 0 Then
                    Cells(currentRow, currentColumn).Interior.Color = RGB(255, 0, 0)
                    upRegCount = upRegCount + 1
                    upRegTot = upRegTot + Cells(currentRow, currentColumn).Value
                End If
                If Cells(currentRow, currentColumn).Value < 0 Then
                    Cells(currentRow, currentColumn).Interior.Color = RGB(0, 255, 255)
                    downRegCount = downRegCount + 1
                    downRegTot = downRegTot + Cells(currentRow, currentColumn).Value
                End If
```

```
            End If
        Next currentColumn

        'Add upRegCount and downRegCount to the last two columns for each row
        Cells(currentRow, lastColumn + 2).Value = upRegCount
        Cells(currentRow, lastColumn + 3).Value = downRegCount
        If upRegCount > 0 Then
            Cells(currentRow, lastColumn + 4).Value = upRegTot / upRegCount
        End If
        If downRegCount > 0 Then
            Cells(currentRow, lastColumn + 5).Value = downRegTot / downRegCount
        End If
        'Reset counts for the next row
        upRegCount = 0
        downRegCount = 0
        upRegTot = 0
        downRegTot = 0
    Next currentRow
End Sub
```

The output data tables after the application of this code may be seen in ***Supplementary Tables 7-10, 13, 14***:

```
Sub conciseTableSigGenesAndLFCValues()
    Dim lastColumn As Long
    Dim lastRow As Long
    Dim currentColumn As Long
    Dim gene As String
    Dim count As Long
    Dim AvgLFC As Long
    Dim tableRowUp As Long
    Dim tableRowDown As Long
    Dim tableCol As Long

    'Find the last column and row with data in the first row
    lastColumn = Cells(1, Columns.count).End(xlToLeft).Column
    lastRow = Cells(Rows.count, lastColumn).End(xlUp).Row
    tableRowUp = 3
    tableRowDown = 3

    'Write table headers
    Cells(2, 8).Value = "Upregulated genes"
    Cells(2, 9).Value = "Datasets gene is upregulated in (n)"
    Cells(2, 10).Value = "Average LFC among upregulated datasets"
    Cells(2, 11).Value = "Downregulated genes"
    Cells(2, 12).Value = "Datasets gene is downregulated in (n)"
    Cells(2, 13).Value = "Average LFC among downregulated datasets"

    'Loop through rows
    For currentRow = 1 To lastRow Step 1
        'Find row with significant Gene and add to up reg table
        If (Cells(currentRow, 2).Interior.Color = RGB(255, 0, 0)) Then
            gene = Cells(currentRow, 1).Value
            count = Cells(currentRow, 2).Value
            tableCol = 8

            Cells(tableRowUp, tableCol).Value = gene
            Cells(tableRowUp, tableCol + 1).Value = count
            Cells(tableRowUp, tableCol + 2).NumberFormat = "0.00"
            Cells(tableRowUp, tableCol + 2).Value = Cells(currentRow, 4).Value
```

```
      tableRowUp = tableRowUp + 1
   End If
   'Find row with significant gene and add to down reg table
   If (Cells(currentRow, 3).Interior.Color = RGB(0, 255, 255)) Then
      gene = Cells(currentRow, 1).Value
      count = Cells(currentRow, 3).Value
      tableCol = 11

      Cells(tableRowDown, tableCol).Value = gene
      Cells(tableRowDown, tableCol + 1).Value = count
      Cells(tableRowDown, tableCol + 2).NumberFormat = "0.00"
      Cells(tableRowDown, tableCol + 2).Value = Cells(currentRow, 5).Value

      tableRowDown = tableRowDown + 1
   End If
Next currentRow
'Find last row to write in Avg, Min, Max and bold each
If (tableRowUp >= tableRowDown) Then
   Cells(tableRowUp, 7).Value = "Average"
   Cells(tableRowUp + 1, 7).Value = "Min"
   Cells(tableRowUp + 2, 7).Value = "Max"

   Cells(tableRowUp, 7).Select
   Selection.Font.Bold = True
   Cells(tableRowUp + 1, 7).Select
   Selection.Font.Bold = True
   Cells(tableRowUp + 2, 7).Select
   Selection.Font.Bold = True

   tableRowDown = tableRowUp
End If

If (tableRowUp < tableRowDown) Then
   Cells(tableRowDown, 7).Value = "Average"
   Cells(tableRowDown + 1, 7).Value = "Min"
   Cells(tableRowDown + 2, 7).Value = "Max"

   Cells(tableRowDown, 7).Select
   Selection.Font.Bold = True
```

```
    Cells(tableRowDown + 1, 7).Select
    Selection.Font.Bold = True
    Cells(tableRowDown + 2, 7).Select
    Selection.Font.Bold = True

    tableRowUp = tableRowDown
End If
'Name table, merge and bold
Cells(1, 8).Value = "Schizophrenia- Analysis for Significant Genes"
Cells(1, 8).Interior.Color = RGB(203, 204, 203)
Range("H1:M1").Select
With Selection
    .HorizontalAlignment = xlCenter
    .VerticalAlignment = xlBottom
    .WrapText = False
    .Orientation = 0
    .AddIndent = False
    .IndentLevel = 0
    .ShrinkToFit = False
    .ReadingOrder = xlContext
    .MergeCells = True
End With
Selection.Merge
Selection.Font.Bold = True
' Bold headers
Range("H2", "M2").Select
Selection.Font.Bold = True
Selection.WrapText = True

Dim total As Double
Dim min As Double
Dim max As Double

'Initialize total and count for up reg
total = 0
count = 0
min = Cells(3, 10).Value
lastRow = Cells(Rows.count, 10).End(xlUp).Row

'Loop through each cell in the row and calculate total, min and max
```

```
For currentRow = 3 To lastRow Step 1
   total = total + Cells(currentRow, 10).Value
   count = count + 1
   If Cells(currentRow, 10).Value > max Then
      max = Cells(currentRow, 10).Value
   End If
   If Cells(currentRow, 10).Value < min & Cells(currentRow, 10).Value > 0 Then
      min = Cells(currentRow, 10).Value
   End If
Next currentRow

'Check if there are numeric values in the column
If count > 0 Then
   'Calculate the average
   Dim average As Double
   average = total / count
End If
‘Set values avg, min and max
Cells(tableRowUp, 10).Value = average
Cells(tableRowUp + 1, 10).Value = min
Cells(tableRowUp + 2, 10).Value = max

'Initialize total and count for down reg
total = 0
count = 0
max = Cells(3, 13).Value
min = 0
average = 0
lastRow = Cells(Rows.count, 13).End(xlUp).Row

'Loop through each cell in the row and calculate total, min and max
For currentRow = 3 To lastRow Step 1
   total = total + Cells(currentRow, 13).Value
   count = count + 1
   If Cells(currentRow, 13).Value > max Then
      max = Cells(currentRow, 13).Value
   End If
   If Cells(currentRow, 13).Value < min Then
      min = Cells(currentRow, 13).Value
   End If
```

```
    Next currentRow

    'Check if there are numeric values in the column
    If count > 0 Then
        'Calculate the average
        average = total / count
    End If
    ‘Set values avg, min and max
    Cells(tableRowUp, 13).Value = average
    Cells(tableRowUp + 1, 13).Value = min
    Cells(tableRowUp + 2, 13).Value = max

End Sub
```
